# Meta-analysis of probability estimates of worldwide variation of CYP2D6 and CYP2C19

**DOI:** 10.1038/s41398-020-01129-1

**Published:** 2021-02-24

**Authors:** Anne B. Koopmans, Mario H. Braakman, David J. Vinkers, Hans W. Hoek, Peter N. van Harten

**Affiliations:** 1 Parnassia Academy, Parnassia Psychiatric Institute, The Hague, the Netherlands; 2 School for Mental Health and Neuroscience, Maastricht University, Maastricht, the Netherlands; 3 Department of Psychiatric Residency Training, Pro Persona Mental Health, Wolfheze, the Netherlands; 4 Department of Criminal Law, Tilburg Law School, Tilburg University, Tilburg, the Netherlands; 5 Department of Psychiatry, University Medical Center Groningen, University of Groningen, Groningen, the Netherlands; 6 Mailman School of Public Health, Columbia University, New York, NY USA; 7 Innova, Psychiatric Centre GGZ Centraal, Amersfoort, The Netherlands

**Keywords:** Scientific community, Clinical genetics

## Abstract

Extensive migration has led to the necessity of knowledge regarding the treatment of migrants with different ethnical backgrounds. This is especially relevant for pharmacological treatment, because of the significant variation between migrant groups in their capacity to metabolize drugs. For psychiatric medications, CYP2D6 and CYP2C19 enzymes are clinically relevant. The aim of this meta-analysis was to analyze studies reporting clinically useful information regarding *CYP2D6* and *CYP2C19* genotype frequencies, across populations and ethnic groups worldwide. To that end, we conducted a comprehensive meta-analysis using Embase, PubMed, Web of Science, and PsycINFO (>336,000 subjects, 318 reports). A non-normal metabolizer (non-NM) probability estimate was introduced as the equivalent of the sum-prevalence of predicted poor, intermediate, and ultrarapid metabolizer CYP2D6 and CYP2C19 phenotypes. The probability of having a CYP2D6 non-NM predicted phenotype was highest in Algeria (61%) and lowest in Gambia (2.7%) while the probability for CYP2C19 was highest in India (80%) and lowest in countries in the Americas, particularly Mexico (32%). The mean total probability estimates of having a non-NM predicted phenotype worldwide were 36.4% and 61.9% for CYP2D6 and CYP2C19, respectively. We provide detailed tables and world maps summarizing clinically relevant data regarding the prevalence of CYP2D6 and CYP2C19 predicted phenotypes and demonstrating large inter-ethnic differences. Based on the documented probability estimates, pre-emptive pharmacogenetic testing is encouraged for every patient who will undergo therapy with a drug(s) that is metabolized by CYP2D6 and/or CYP2C19 pathways and should be considered in case of treatment resistance or serious side effects.

## Introduction

Migration is a growing global phenomenon so that Western-trained psychiatrists are increasingly treating migrants with different cultural and ethnic backgrounds^1^. In the psychopharmacological treatment of migrants, variation in drug metabolism is an important aspect that must be taken into account^2^. In psychiatry, CYP2D6 and CYP2C19 are important drug-metabolizing enzymes^3–8^. For example, drugs that are metabolized by CYP2D6 include first- and second-generation antipsychotics, selective serotonin receptor inhibitors, and tricyclic antidepressants^6^. Among those metabolized by CYP2C19 are benzodiazepines, selective serotonin receptor inhibitors, and tricyclic antidepressants^6^. Individuals’ genetic variation is the most important factor influencing the kinetics of drug metabolism and thus may contribute to intolerability-related discontinuation or treatment failure^9^.

The outcome of a pharmacogenetic test (i.e., a patient’s genotype, sometimes also referred to as diplotype) can be translated into a predicted phenotype. A combination of functional and non-functional alleles is responsible for the activity of the enzymes. There are four phenotype groups: poor (PM), intermediate (IM), normal (NM) (previously referred to as “extensive”), and ultrarapid metabolizers (UM), which are used to predict whether and how well a drug is metabolized. The same drug dosage may lead to a higher plasma level in PMs and IMs, compared to NMs, because of slower drug clearance, while UMs may have lower plasma levels than NMs because of a higher rate of drug clearance. Plasma levels are often related to the efficacy of a drug and the risk of dose-related side effects, with more severe side effects found in PMs and IMs than in NMs^10–15^.

The prevalence of *CYP* polymorphisms also varies considerably across ethnic groups and plays a major role in inter-individual and inter-ethnic differences in drug metabolism and response^16^. For example, in European populations, just 2–3% of the population have a CYP2D6 UM profile, compared to 20–29% in East-African populations^17,18^. In contrast, CYP2C19 PMs are considerably more frequent in Asians (~12%) than in Europeans (~2%)^19^. Some allelic variants such as CYP2D6*40 and *45 are only seen in specific populations^20,21^.

The Clinical Pharmacogenetics Implementation Consortium (CPIC) has published guidelines with recommendations for drug choice and dosage based on phenotype predictions^22–25^.

Other groups, including the Royal Dutch Association for the Advancement of Pharmacy—Pharmacogenetics Working Group, have also published guidelines (information for both organizations is available through the Pharmacogenomics Knowledge Base at https://www.pharmgkb.org/guidelines). *CYP2D6* and *CYP2C19* allele frequency information has been compiled by CPIC and is available at https://www.pharmgkb.org/page/cyp2d6RefMaterials. Gaedigk et al., Fricke-Galindo et al., and Llerena et al. have described CYP2D6 and CYP2C19 phenotype prediction from genotype across world populations^17,19,26^.

Although there is a wealth of information, pharmacogenetics is still not being widely used in clinical practice. Several studies have shown the relationship between CYP activity, blood serum levels, and side effects, but there have been few studies on clinical effectivity. Most of the studies are cross-sectional and observational, while prospective studies are often underpowered^4,27–29^. For some drugs, clinicians are used to working with therapeutic drug monitoring and they may prefer this over genotyping.

Another reason is the lack of education of practitioners on this topic and the belief that pharmacogenetics “is not ready” for use in daily clinical practice^30–33^. Despite these barriers, pharmacogenetics is increasingly being adopted by major health centers, and the body of literature in support of pharmacogenetic testing is growing^27,29,34–39^.

The aim of our meta-analysis was to assess studies reporting clinically useful information about *CYP2D6* and *CYP2C19* genotype frequencies across populations and ethnic groups worldwide. We introduce the concept of the non-normal metabolizer (non-NM) probability estimate, for which we calculated the sum-prevalence of a population for having a poor, intermediate, or ultrarapid CYP2D6 or CYP2C19 predicted phenotype. The sum-prevalence of these three predicted phenotypes presents a single measurement for non-normal metabolism in the populations of interest. It is defined as the equivalent of the prevalence of PM + IM + UM predicted phenotypes of the enzyme in percentages.

## Methods

For this study, we followed the checklist in the Preferred Reporting Items for Systematic Reviews and Meta-Analyses statement^40,41^. The protocol for the current systematic review was not registered prior to the review.

### Review of literature

We conducted a literature review using the Embase, PubMed, Web of Science, and PsycINFO databases (1990–2019). The terms “CYP2D6” AND/OR “CYP2C19” AND “prevalence” OR “ethnicity” OR “race” AND “healthy subject” OR “normal control,” and variations on these terms and the names of different countries and continents were used in all fields. The last search was conducted on July 3, 2019.

Our inclusion criteria were: (1) *CYP2D6* or *CYP2C19* allele frequencies from original data were reported; (2) the evaluated subjects did not have a specific disease (controls from case–control studies were included); (3) ethnicity was reported; (4) the article was published in English; (5) a minimum of 20 participants was investigated. If only an abstract was available, the article was included if all the above information was available; and (6) in order to be able to calculate a probability estimate, it was necessary to assess a minimum number of non-functional alleles and alleles with decreased function as well as a number of gene duplications. The genotyping assay included at least the following allelic variants: CYP2C19*2 and *17 in Africans, Americans, Europeans, Middle Easterners, Central/South East-Asians and African Americans; CYP2C19*2, *3, and *17 in Oceanians and CYP2C19*2 and *3 in East Asians; CYP2D6*2, *5, *17, *29, and *41 in Africans; CYP2D6*2 and *4 in Americans; CYP2D6*2 and *10 in East Asians; CYP2D6*2, *4, and *41 in Europeans; CYP2D6*2, *4, *10, and *41 in Middle Easterners and Central/South East-Asians; CYP2D6*5 in Oceanians; and CYP2D6*2, *4, *17, and *29 in African Americans.

An eligibility assessment was performed independently in a standardized manner by A.B.K. and D.J.V. The first screening was based on the article abstracts; the next selection was based on the full text. Disagreements between the two reviewers were resolved by discussion to reach a consensus.

### Data extraction

The data were independently extracted from the studies by two investigators (A.B.K. and P.B.) and randomly checked by two investigators (D.J.V. and A.B.K.). For some studies, authors were contacted for clarification of the data.

Information was extracted from each study as follows: (1) ethnicity of the participants; (2) definition of ethnicity (self-reported or genetic ancestry); (3) country of the studied population; (4) number of study participants; (5) study design (prevalence study, case–control study, experimental study); (6) allele frequencies; (7) diplotype frequencies; (8) predicted phenotype frequencies.

Star (*) alleles were assigned according to the Pharmacogenetic Variation (PharmVar) Consortium at PharmVar.org^42,43^.

### Ethnicity and geographical regions

To be able to compare outcomes with previous meta-analyses, reported ethnicity was assigned to geographic regions, as done in previous meta-analysis about this subject, according to the Human Genome Diversity Project^44^. Seven major regions were considered: Africa, Americas (including Latino Americans and indigenous inhabitants of North America and Canada), East Asia, Europe (including North Americans and Canadians), the Middle East, Oceania, and Central/Southeast Asia; with one exception, namely, that African Americans were listed separately from Africans^17^. Here the frequencies of PM, IM, normal metabolizer (NM), and UM are reported by ethnicity, whereas the probability estimates of being a non-NM are reported by country. In many studies, these two factors—country and ethnicity—overlap, but for some studies we had to assign an ethnicity to a country to be able to show the information in world maps (i.e., the two factors were not distinguished). The origin of the investigated ethnicity determined the country and region to which a population was assigned. An exception was made for Latin America, in which the population is an admixture of multiple origins (e.g., European, African, Asian, and Amerindian) and no clear lineages can be determined; they were all considered as populations of the Americas and determined as belonging to the country they live in^45,46^. For some ethnicities, we could not determine a country of origin (for example, East Asians or Europeans) so we have indicated them as “missing” in the figures.

### Translation of genotype into phenotype

For each geographical region, the mean frequency of alleles was determined. In order to predict CYP2D6 and CYP2C19 phenotype frequencies from genotype data, we applied the activity score (AS) system to both genes (the AS system is widely used for CYP2D6 and was adapted to CYP2C19 to facilitate the translation process)^47^. Briefly, a normal function allele was valued as 1, decreased function alleles as 0.25 or 0.5, a non-functional allele as 0, and increased function allele as 1.5. Gene duplications received double the value of their singleton counterparts. Homozygous carriers of non-functional alleles were classified as PMs (AS = 0). Carriers with one functional or decreased function allele and one non-functional allele and those carrying two decreased function alleles were classified as IMs (AS = 0.25–1)^47^. Homozygous carriers of normal function alleles and heterozygous carriers with one decreased function and one normal function allele were classified as NMs (AS = 1.25–2.25)^47^. Carriers of one or more increased function alleles and carriers of a duplication or multiplication of a functional allele were classified as UMs (AS > 2.25)^47^ (https://cpicpgx.org/resources/term-standardization/). CYP2C19 rapid and ultrarapid metabolizers were pooled and analyzed as UMs. The functionality of the *CYP2D6* and *CYP2C19* alleles was classified as listed by PharmVar in Table 1.

**Table 1:** Functionality of *CYP2D6* and *CYP2C19* alleles (https://www.pharmvar.org/gene).

	*CYP2D6*	*CYP2C19*
0	*3–*8, *15, *18, *31, *36, *47, *51, *56, *57, *62, *92, *100, and *101	*2, *3, *4, *5, *6, *7, *8, *23, and *24
0.25–0.5	*9, *10, *17, *29, *41, *49, *50, *54, *55, *59, and *72	*9, *10, *12, *16, *25 and *27
1	*1, *2, *27, *39, *45, *46, and *48	*1, *13, *15 and *18
1.5	*53	*17
Unknown	*43, *60, *65, *82, *84, *85, and *86	

In this meta-analysis, we applied strict criteria. To maximize the accuracy of the frequencies of the predicted phenotypes, we only predicted a phenotype if the original publication reported a minimum of non-/decreased function alleles and the assays included tests for gene duplications. Since the prevalence of alleles differed greatly per region, we used criteria specific for each geographical region. Alleles more prevalent than 0.05 (5%) in the major region (Table 2) had to be investigated in the countries within that region to be included in the phenotype predictions.

**Table 2:** CYP2D6 and CYP2C19 allele frequencies per major geographical region^a^.

Region		CYP2D6*2	CYP2D6*4	CYP2D6*5	CYP2D6*10	CYP2D6*17	CYP2D6*29	CYP2D6*35	CYP2D6*41	CYP2D6*45	CYP2C19*2	CYP2C19*3	CYP2C19*17
Africa	*M*	**0.25**	**0.10**	0.04	0.03	**0.19**	**0.09**	0.01	0.04	**0.07**	**0.17**	0.00	**0.23**
	*n*	6815	7012	6796	6711	6631	5941	181	6206	153	9140	7189	5477
	SD	0.07	0.03	0.02	0.01	0.03	0.02	0.01	0.04	0.03	0.03	0.01	0.02
African Americans	*M*	**0.20**	**0.08**	0.04	0.04	**0.19**	**0.06**	0.00	0.03	0.03	**0.18**	0.00	**0.22**
	*n*	3911	4057	4057	3811	3811	3647	131	3648	252	3877	3877	3533
	SD	0.03	0.01	0.01	0.01	0.01	0.00	0.00	0.02	0.00	0.01	0.00	0.01
Americas	*M*	**0.25**	**0.12**	0.03	0.02	0.02	0.01	0.02	0.04	0.00	**0.11**	0.00	**0.13**
	*n*	18,065	19,802	18,571	18,580	16,018	15,496	8399	16,052	528	12,261	11,155	9091
	SD	0.08	0.05	0.02	0.03	0.03	0.02	0.02	0.03	0.00	0.04	0.01	0.04
Central/South East Asia	*M*	**0.34**	**0.10**	0.03	**0.10**	0.00	0.00	0.00	**0.13**		**0.33**	0.01	**0.15**
	*n*	10,523	12,393	10,573	11,180	9797	8700	10	8822		14,789	13,076	10,837
	SD	0.07	0.04	0.03	0.11	0.00	0.00	0.00	0.02		0.05	0.03	0.04
East Asia	*M*	**0.13**	0.00	**0.05**	**0.49**	0.00	0.00	0.00	0.02		**0.30**	**0.07**	0.02^b^
	*n*	16,297	15,510	18,049	18,978	11,050	5234	3039	14,608		24,120	24,861	6756
	SD	0.03	0.01	0.01	0.08	0.00	0.00	0.00	0.01		0.05	0.03	0.01
Europe	*M*	**0.36**	**0.18**	0.03	0.02	0.00	0.00	**0.06**	**0.08**		**0.15**	0.00	**0.23**
	*n*	128,397	143,492	134,524	127,590	125,502	123,554	1040	125,303		180,615	130,396	170,889
	SD	0.08	0.04	0.01	0.02	0.01	0.01	0.01	0.03		0.02	0.00	0.02
Middle East	*M*	**0.29**	**0.09**	0.02	**0.14**	0.03	0.02		0.25		**0.15**	0.02	**0.25**
	*n*	1123	2902	1424	1816	1116	340		961		4768	3728	1903
	SD	0.11	0.06	0.01	0.11	0.03	0.01		0.09		0.08	0.04	0.06
Oceania	*M*	**0.05**	0.04	**0.05**	0.02	0.00	0.00		0.02		**0.77**	**0.14**	**0.13**
	*n*	279	361	361	361	301	62		122		5687	149	24
	SD	0.04	0.05	0.03	0.02	0.00	0.00		0.02		0.09	0.15	0.00
Total	*M*	**0.32**	**0.15**	0.03	**0.07**	0.01	0.01	0.02	0.07	0.02	**0.18**	0.01	**0.21**
	*n*	185,410	205,529	194,355	189,027	174,226	162,974	12,800	175,722	933	255,257	194,431	208,510
	SD	0.11	0.06	0.02	0.15	0.05	0.02	0.02	0.04	0.03	0.11	0.03	0.05

Bold = prevalence >0.05.

*M* mean, *n* number of genotyped subjects, *SD* standard deviation.

^a^For references, please see Supplemental Table 1.

^b^For this specific region, CYP2C19*17 is not part of the minimum set of alleles for reliable phenotype prediction, because with 2% it is less prevalent than 5% in this area. This means that studies from East Asia who only determined CYP2C19*2 and *3 are included in the meta-analysis.

### Calculations and statistics

All analyses were performed with IBM SPSS Statistics Version 25. If only diplotypes were reported, single allele frequencies were calculated. If only single allele frequencies were reported, diplotype frequencies were calculated using the Hardy–Weinberg equilibrium (*p*^2^ + 2*pq* + *q*^2^ = 1). For studies that did not report the prevalence of CYP2D6*1, the allele frequency was calculated as 100% minus the sum of variants^17^. The diplotypes were translated into predicted phenotypes according to the CPIC.

We introduce here the concept of the non-NM probability estimate. It is defined as the sum of the prevalence of PM + IM + UM predicted phenotypes of the enzymes CYP2D6 or CYP2C19 in percentages. Thus it is equivalent to the prevalence (as percentage) of all the non-normal phenotypes in a population. We use the term probability estimate exclusively in this sense and it is in fact a proportion of the possible outcomes in a population. It is equal to 100% minus the percentage of NM in a given population.

The studies were weighted by sample size (number of participants) when we calculated the mean predicted phenotypes per country and ethnicity.

## Results

**Fig. 1:**
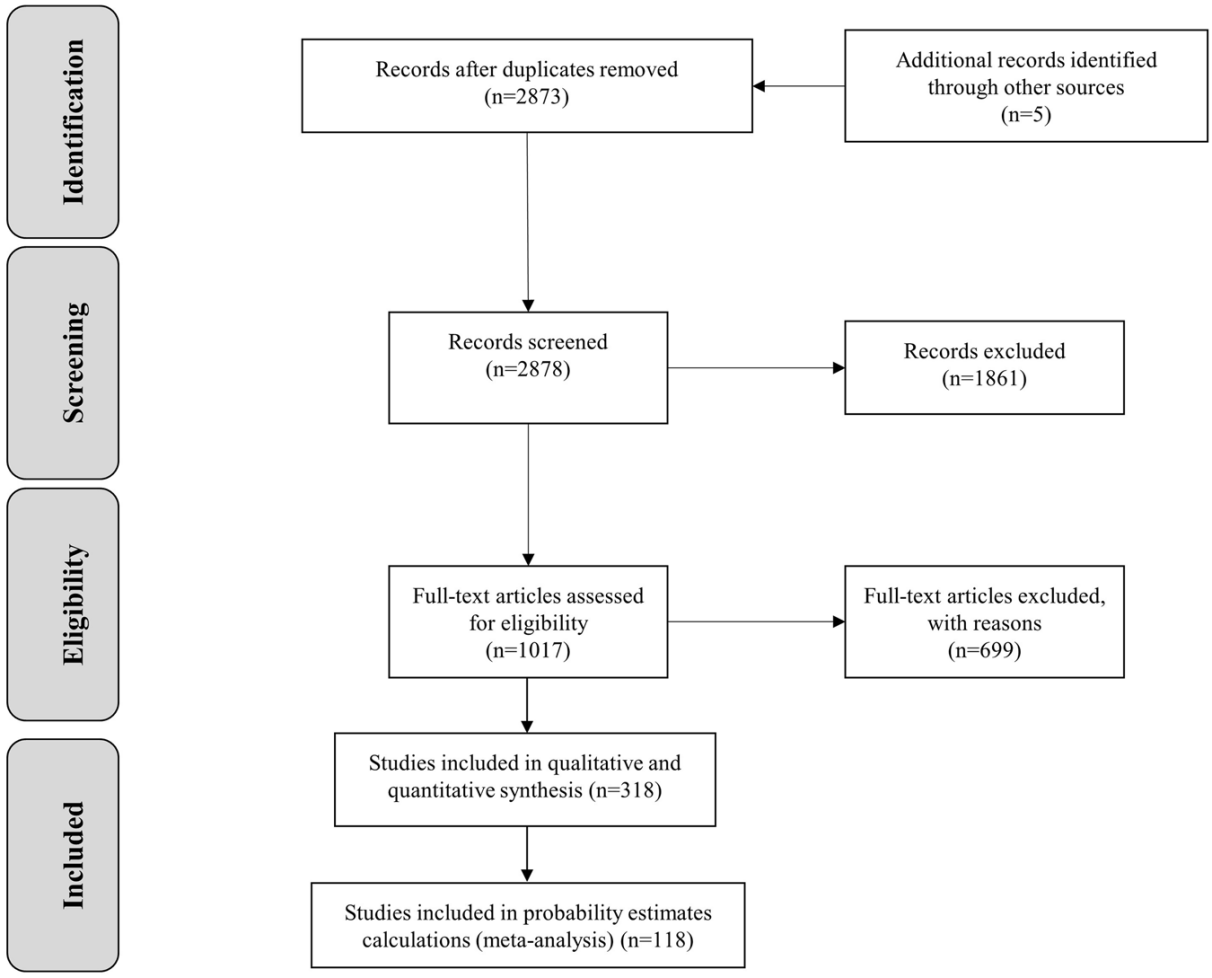
PRISMA flow diagram of the studies included in the analyses. n = number of studies.

Of the 2873 publications retrieved from the database, 318 original research papers met our inclusion criteria (Fig. 1). The analyses of CYP2D6 (*n* = 200 papers) and CYP2C19 (*n* = 159 papers) included 261,296 and 257,745 healthy individuals. The alleles most frequently investigated were CYP2D6*1–*6, *10, *17, and CYP2C19*1–*3. Allele frequencies are shown per major geographical region in Table 2 (for references, please see Supplemental Table 1). The most frequently observed variant alleles across all subjects were CYP2D6*2, *4, *10, and *41 and CYP2C19*2 and *17. As expected, allele frequencies varied substantially among ethnicities and countries. We found 89 studies that reported on more than one ethnic group. Overall, African and Middle Eastern countries were underrepresented, while European populations were the most frequently investigated.

### CYP2D6

#### Prevalence of predicted phenotypes by ethnicity

Predicted phenotype was reported or could be inferred from 51 studies for 116 ethnicities, covering *n* = 194,714 individuals. These studies were selected for fulfilling the minimum number of alleles tested as prescribed by our region-specific criteria. Due to the high frequency of allele duplications, high percentages of CYP2D6 UM were found in the Mozabite people, a Berber ethnic group in the Sahara, North Africa (39.5%)^48^, in non-Austronesian Melanesians (21.5%)^48^, and in the ethno-religious Druze from the Middle East (21.4%)^48^. High percentages of CYP2D6 PM were found in Europeans, for example, in the British (12.1%)^49^, the Danish (10.6%)^50^, and Basque (French) people (9.7%)^48^ due to the high frequency of CYP2D6*4. Frequencies of CYP2D6 predicted phenotypes by ethnicity are summarized in Table 3 (for references, please see Supplemental Table 2).

**Table 3:** Mean frequencies of CYP2D6 predicted phenotypes per ethnicity^a^.




*M* mean, *n* number of genotyped subjects, *SD* standard deviation, *PM* poor metabolizers, *IM* intermediate metabolizers, *NM* normal metabolizers, *UM* ultrarapid metabolizers.

^a^For references, please see Supplemental Table 2.

^b^An SNP combination that could not be assigned to a known allele/phenotype.

#### Probability estimates by country

**Fig. 2:**
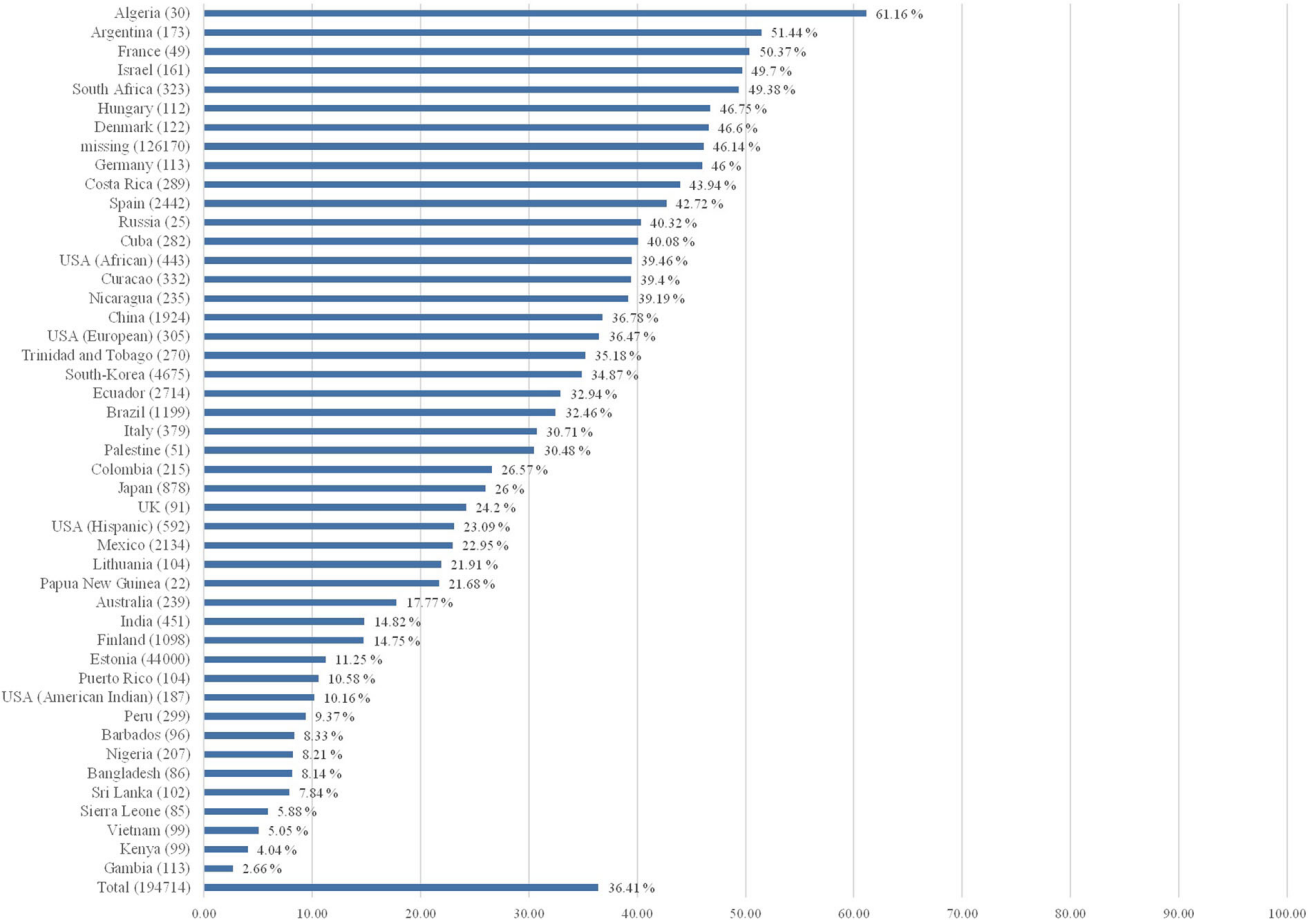
CYP2D6 non-normal probability estimate per country. Country (genotyped subjects); non-normal probability estimate in percentage.

**Fig. 3:**
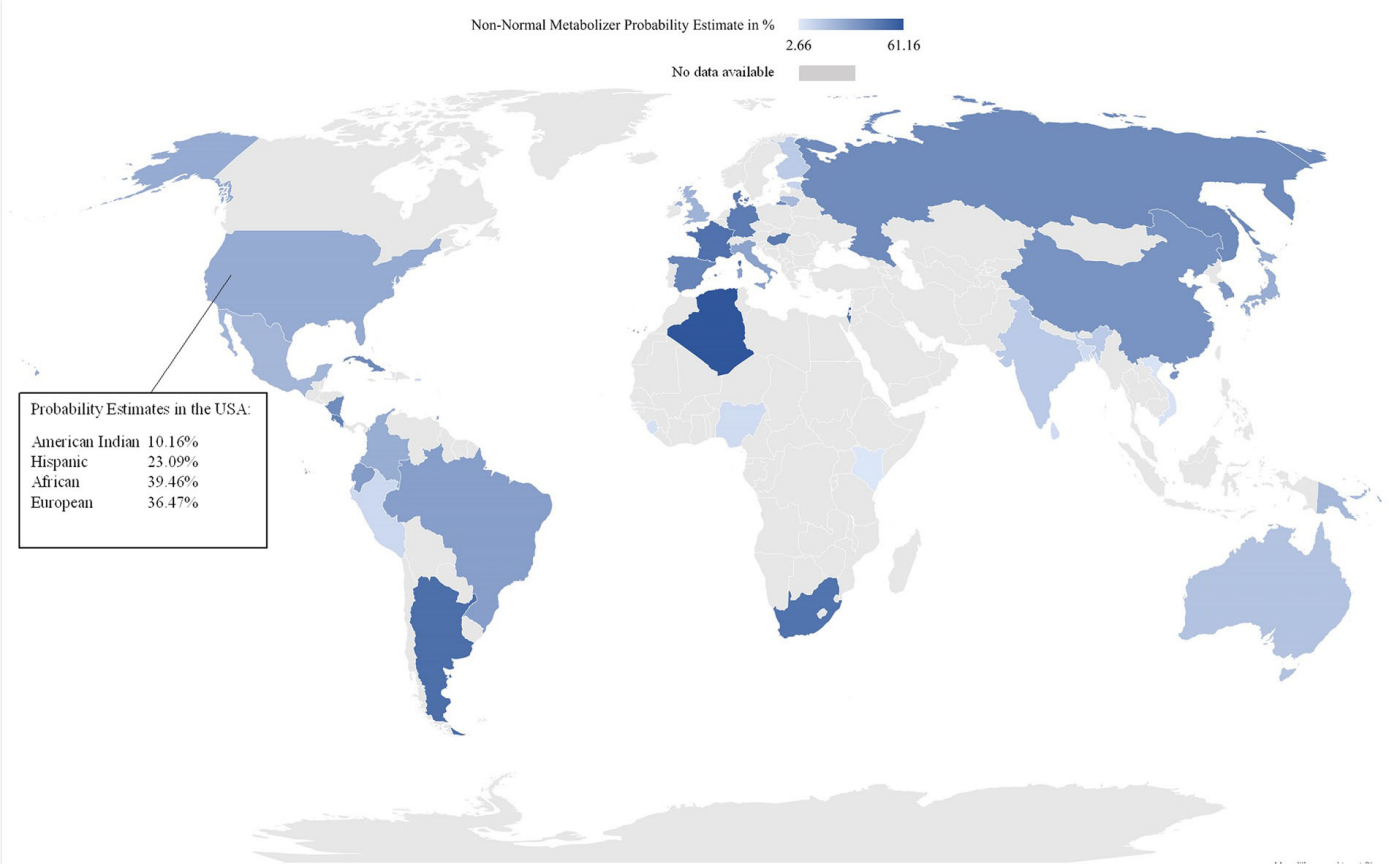
Worldwide CYP2D6 non-normal metabolizer probability estimates.

The probability of having a CYP2D6 non-NM predicted phenotype is the highest in Algeria (non-NM probability estimated to be 61.2%; *4, *17, *41, and duplications)^48^, Argentina (non-NM probability estimate 51.4%; *4, *41, and duplications)^51^, and France (non-NM probability estimate 50.4%; *4, *5, *41, and duplications)^48^. The CYP2D6 non-NM probability estimate was lowest in several populations from Africa (Gambia 2.7%, Kenya 4.0%, and Sierra Leone 5.9%) and South-East Asia (Vietnam 5.1%, Sri Lanka 7.8%)^49^. See Fig. 2 for CYP2D6 non-NM probability estimates and Fig. 3 for CYP2D6 non-NM probability estimates plotted on a world map.

### CYP2C19

#### Prevalence of predicted phenotypes by ethnicity

Data were retrieved from 73 studies describing 225,173 subjects of 85 ethnic groups/countries. These studies were selected for fulfilling the minimum number of alleles tested as prescribed by our region-specific criteria. The UM predicted phenotype is rather common in Ecuadorian Mestizos (41.4%)^52^, Dargins (39.8%)^53^, and ethnic groups living in the North Caucasus in Russia and in Burushu, Pakistan (39.0%)^54,55^ owing to a high prevalence of >20% of CYP2C19*17 in all these populations. High percentages of CYP2C19 PMs were found in Indian and Pakistani populations (Naik 31.0%^56^ and Saraiki 20.0%^55^), Tohoku Japanese (18.9%)^57^, and in Chinese Hui (28.0%)^58,59^ due to the presence of the CYP2C19*2 and *3 non-functional alleles. Table 4 summarizes the frequencies of CYP2C19 predicted phenotypes by ethnicity (for references, please see Supplemental Table 3).

**Table 4:** Mean frequencies of CYP2C19 predicted phenotypes per ethnicity^a^.




*M* mean, *n* number of genotyped subjects, *SD* standard deviation, *PM* poor metabolizers, *IM* intermediate metabolizers, *NM* normal metabolizers, *UM* ultrarapid metabolizers.

^a^For references, please see Supplemental Table 3.

^b^An SNP combination that could not be assigned to a known allele/phenotype.

#### Probability estimates by country

**Fig. 4:**
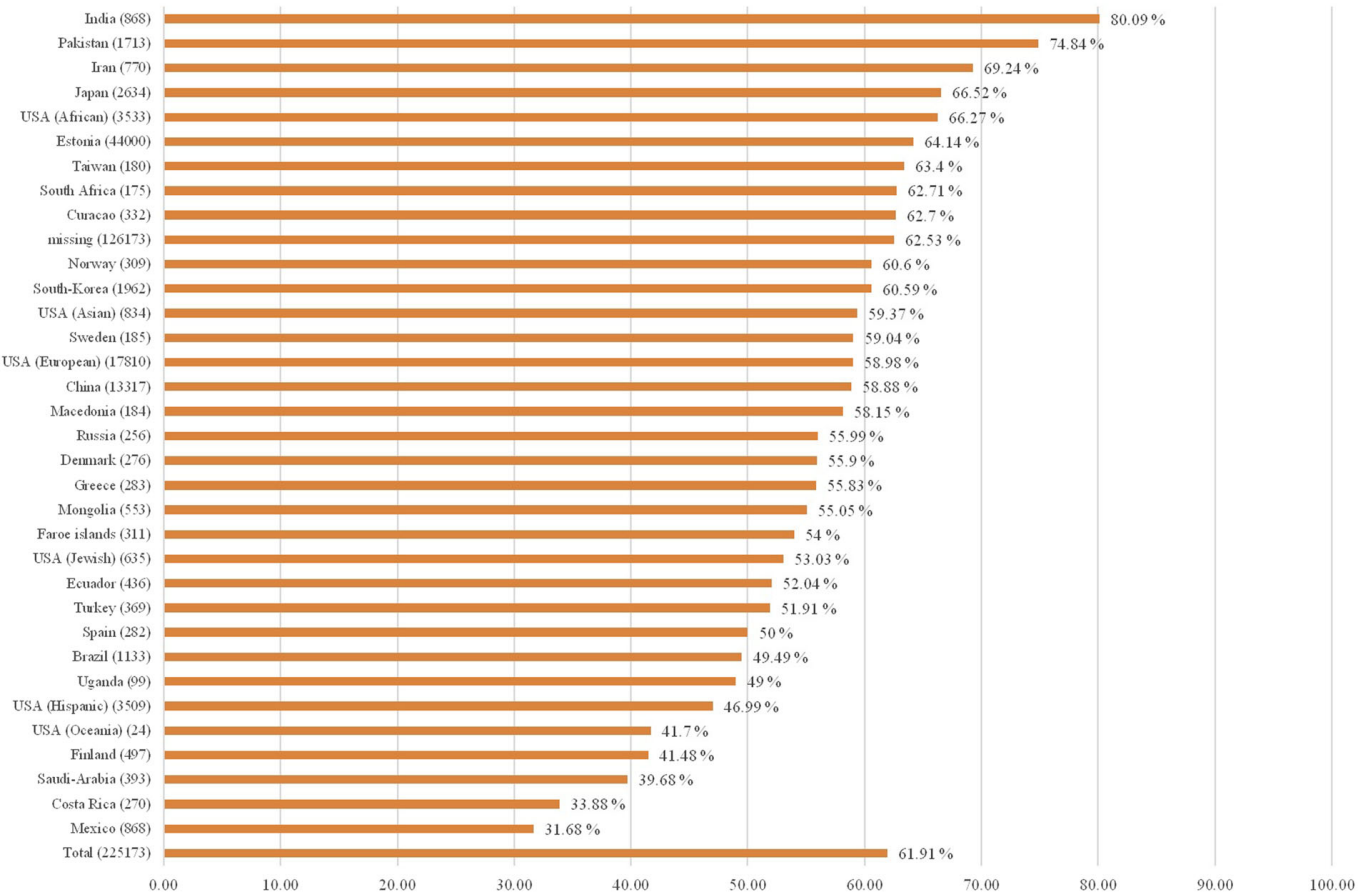
CYP2C19 non-normal probability estimates per country. Country (genotyped subjects); non-normal probability estimate in percentage.

**Fig. 5:**
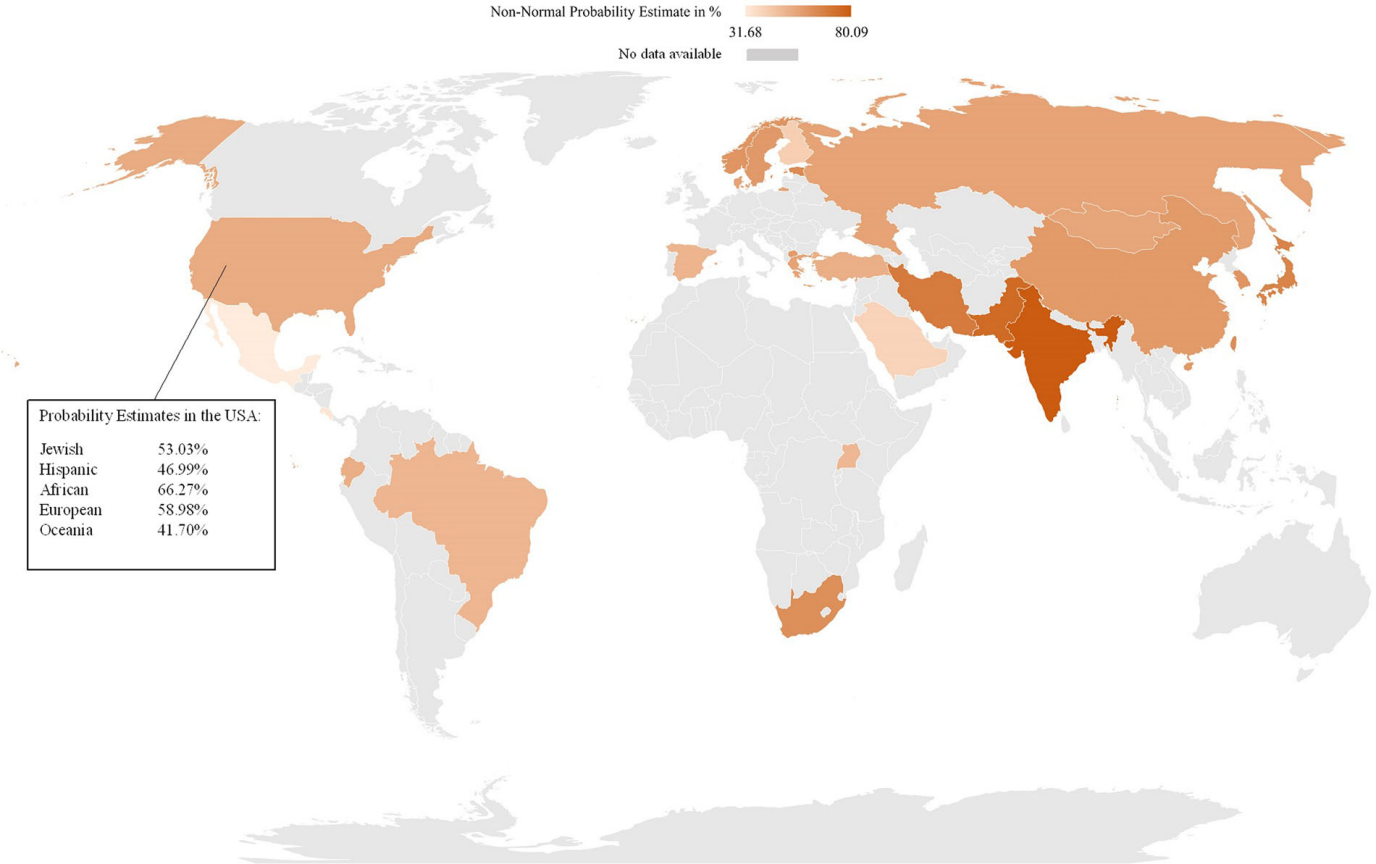
Worldwide CYP2C19 non-normal metabolizer probability estimates.

The probability of having a CYP2C19 non-NM predicted phenotype due to high frequencies of the non-functional CYP2C19*2 allele and/or the increased function CYP2C19*17 allele is highest in India (non-NM probability estimate 80.1%)^56,60,61^, Pakistan (non-NM probability estimate 74.8%)^55,62,63^, and Iran (non-NM probability estimate 69.2%)^64,65^. The probability is lowest in countries in the Americas, particularly Mexico (non-NM probability estimate 31.7%)^66–68^ and Costa Rica (non-NM probability estimate 33.9%)^69^. CYP2C19 non-NM probability estimates are shown in Fig. 4, and Fig. 5 displays CYP2C19 non-NM probability estimates on a world map.

## Discussion

In this meta-analysis, we introduce a clinical useful concept of a non-NM probability estimate as the equivalent of the sum-prevalence (in percentages) of PM + IM + UM predicted CYP2D6 and CYP2C19 phenotypes. The mean totals of non-NM probability estimates worldwide were 36.4% (CYP2D6) and 61.9% (CYP2C19). This means that more than half of all psychiatric patients have a non-normal predicted CYP2D6 and/or CYP2C19 metabolizer phenotype. Since >75% of the psychopharmacological drugs are metabolized by one or both of these enzymes^6,70^, the results of our meta-analysis emphasize the importance of integrating pharmacogenetic information into clinical practice, especially when treating patients who have had adverse drug events or show treatment resistance.

We included a total of 318 studies in our meta-analysis of *CYP2D6* and *CYP2C19* genotypes in healthy populations of which genotype requirements were fulfilled by 118 studies (37%). This is an important requirement, because having too few *CYP2D6* or *CYP2C19* alleles in a study may not accurately determine predicted phenotype from genotype data.

### Diversity within major regions

Although grouping populations can simplify reporting of pharmacogenetic alleles, we grouped ethnicities within geographical regions to enable comparisons with other published meta-analyses^71,72^. The prevalences of our CYP2D6 predicted phenotypes were in general agreement with those reported by Gaedigk et al.^17^. However, our data predicted a higher percentage of UMs for Africans (i.e., 5.5%, *n* = 562 versus 3.8%, *n* = 430). This difference may be explained by the fact that we weighted the number of genotyped subjects when calculating the mean allele frequencies for our meta-analysis. In Gaedigk et al.’s report, studies were not weighted by sample size, so small studies might have had a bigger influence on the mean. We also included two studies in Africans that were not included by Gaedigk et al. due to their small sample size and thus not meeting their requirements of at least 50 study subjects; they both reported higher frequencies of UMs^48,73^. In contrast, in the South Central Asian population the percentage of UMs found by Gaedigk et al. was higher than those reported here, 2.8% (*n* = 370) vs. 2.1% (*n* = 434), due to the inclusion of two studies in South East Asians in this meta-analysis, which found no UMs^73,74^.

In contrast with Fricke-Galindo et al.^19^ and the CPIC’s *CYP2C19* allele frequency table (https://www.pharmgkb.org/page/cyp2c19RefMaterials), we found a much higher prevalence of 25.0% for CYP2C19 UMs in Oceania (vs. 0% and 1.5%). This is because we only included studies investigating the CYP2C19*17 increased function allele, which leads to rapid and ultrarapid metabolizer predicted phenotypes. Studies that do not investigate this allele yield underestimates of the prevalence of UMs.

### Clinical practice

Some issues need to be addressed in translating genotype data to information useful for clinical practice. Although prevalences of non-NM are high, there is no conclusive evidence whether *CYP* genotyping is beneficial for clinical outcomes in psychiatric practice. There are a few prospective studies analyzing the clinical utility of *CYP* genotyping and they report contradictory outcomes in diverse populations^27–29^. So far, it is still unclear which patient groups might benefit from genotyping and see better treatment outcomes. One reason for the uncertainty is the possibility of the transformation of genotypic EMs into phenotypic PMs by multiple causes, for example, due to smoking, CYP2D6 and CYP2C19 inhibiting medication (e.g., bupropion and esomeprazole), CYP2D6 and CYP2C19 inducing medication (e.g., oritavancin and carbamazepine), and inflammation or co-morbidities^75,76^. This phenomenon is called phenoconversion: it has been described in diverse populations^77–79^. If this is happening on a large scale, it means genotypic outcomes could be unreliable for use in clinical practice. It may already influence outcomes of studies on the effectivity of *CYP* genotyping in clinical practice^28^. Another issue may arise with genotyping patients who have been on treatment for several years, because their brain has adapted to the changed levels of neurotransmitters and the side effects are no longer reversible^80,81^.

Lastly, the category of the IM has been the subject of debate^82^. In this meta-analysis, we categorized the IM as defined by the CPIC^47^. Because IMs only show minor differences in metabolism from NMs^83^, one could categorize them in the NM group. However, other studies have indicated that IMs show lower oral drug clearance, higher blood serum levels, and have higher chances of side effects than NMs^84–86^. For this reason, we consider that the IM status is clinically relevant for psychiatric patients and guidelines for some medications are now advising dose adjustments for this predicted phenotype^25,87^. Estimations of the worldwide prevalence of only PM + UM were 7.75% (CYP2D6) and 32.94% (CYP2C19) (Supplemental Figs. 1 and 2).

### Strengths and limitations

We included studies with a small number of participants (*n* ≥ 20), as well as controls from case–control studies, which increased the number of studies we could include. Bias was minimized by excluding studies of populations diagnosed with a disease to prevent confounding our data (i.e., disease associations with specific allele frequencies^88–94^). On the other hand, some large studies had to be excluded due to their inclusion of non-healthy individuals, which might have biased the outcomes of some countries^95^.

Another strength is that we used region-specific inclusion criteria to maximize the accuracy of the phenotype predictions^17,19,26^. This helped to avoid applying criteria based on studies in Western countries to other regions of the world. This led to the exclusion of studies reporting on too few allelic variants and of studies focusing on the determination of only PM or UM in a population^18,96,97^.

Studies reporting allele frequencies of merely CYP2C19*2 and *3 or studies investigating *CYP2D6* allele duplications, but with no minimum set of variants, are certainly of scientific importance but not of practical importance for clinicians because no complete risk inventory of the metabolizer phenotype could be determined. Because we excluded studies not investigating CYP2C19*17, we had only one report describing Oceanians (*n* = 24)^98^, which did not identify any PMs.

A limitation is that we were depended on the sensitivity of the tests of the individual studies. For example, because of overlap in single-nucleotide polymorphisms in the CYP2D6*10 and *36 and in the CYP2D6*17 and *40 allele, a slight overestimation or underestimation of some predicted phenotypes might have been reported in some studies^99^.

The inclusion of studies with a small number of participants (20–50) could have led to an overestimation or underestimation of predicted phenotypes in some populations, but the influence on the mean prevalence was minimized by weighting the number of genotyped subjects. We may still have made overestimates or underestimates where there are few studies for a certain region/country along with a relatively small number of studied subjects. Because few studies reporting specific minority ethnicities met our inclusion criteria, we did not want to exclude potentially valuable information from our meta-analysis by setting too-stringent participant number requirements.

Although we only included studies on homogenous ethnic groups in this meta-analysis, we are aware of the limitations of grouping ethnicities based on self-reported ethnicity. Although ancestry based on genetic information is more objective than self-reported ethnicity, much of the research into *CYP* genotypes has been based on self-reported ethnicity, while for a few minority populations, some genetic data were systematically analyzed. In a study of 103,006 participants with 23 ethnicities, a very high correspondence was found between self-reported ethnicity and genetic ancestry^100^. Only African Americans and Latino Americans demonstrated a higher degree of ancestral admixture than self-reported.

Second, although studies of genetic ancestry show there is a strong linkage between belonging to an ethnic group and coming from a certain geographical region, ethnicity is not always the same as geographical region^44^. Ethnic groups migrate, and although some ethnicities show almost no admixture with the local ethnicity even many years after migration, other ethnicities do show a mixture of multiple ancestors.

Especially in countries in the Americas, North America, and Canada, ethnic backgrounds can be diverse and individual ethnicity is increasingly blurred by admixture, making self-reported ethnic background or geographical location less predictive for a correct estimation on a non-NM predicted phenotype^76^. The probability estimates per country (Figs. 2–5) are means of the probability estimates of these different ethnicities and must therefore be interpreted with caution.

The total means are mean probability estimates of all the included populations and represent a worldwide mean probability estimate. Because countries were not weighted by the number of inhabitants, small countries with large study populations have a relatively large influence on the estimated mean. In addition, some geographical regions were significantly under-investigated (Africa and the Middle East) and their predicted phenotype distributions are not adequately represented in the total estimated means.

## Conclusions

In this comprehensive meta-analysis of worldwide CYP2D6 and CYP2C19 genotype variation, (>336,000 subjects, 318 reports), we found that the mean total probability estimates for a non-NM predicted phenotype are 36.4% for CYP2D6 and 61.9% for CYP2C19. The estimates reveal a large geographical variation (3–61% and 32–80%, respectively). Our results suggest that more than half of the world population has a non-normal CYP2D6 and/or CYP2C19 metabolizer predicted phenotype. Based on the documented probability estimates, pre-emptive pharmacogenetic testing is encouraged for every patient who will undergo therapy with a drug(s) that is metabolized by CYP2D6 and/or CYP2C19 pathways and should be considered in case of treatment resistance or serious side effects.

Second, many of the studies were not relevant for clinical practice, because they only investigated a minimum number of allelic variants and thus any phenotype prediction is unlikely to be accurate. Especially when estimating the prevalence of the CYP2C19 UM predicted phenotype, studies in all regions except for East Asia should genotype on *17 to come to a reliable phenotype prediction. We therefore recommend that, when allele frequencies are being studied, a minimum number of alleles—depending on the geographical region—must be assessed to be able to predict phenotypes as accurately as possible^101^.

## Supplementary information

SUPPLEMENTAL FIGURE AND TABLE LEGENDS

Supplemental Table 1: References for Table 2

Supplemental Table 2: References for Table 3

Supplemental Table 3: References for Table 4

Supplemental Figure 1. Prevalence of CYP2D6 PM+UM per country

Supplemental Figure 2. Prevalence of CYP2C19 PM+UM per country
