# Supplementary material for: Meta-analysis of probability estimates of worldwide variation of CYP2D6 and CYP2C19
Source: Transl Psychiatry. 2021 Feb 24;11:141. doi: 10.1038/s41398-020-01129-1 (PMC7904867; doi:10.1038/s41398-020-01129-1)
Supplement: Supplementary file 1 — SUPPLEMENTAL FIGURE AND TABLE LEGENDS [file 41398_2020_1129_MOESM1_ESM.docx]

**SUPPLEMENTAL FIGURE AND TABLE LEGENDS**

**Supplemental Figure 1. Prevalence of CYP2D6 PM+UM per country**

**Supplemental Figure 2. Prevalence of CYP2C19 PM+UM per country**

**Supplemental Table 1: References for Table 2**

**Supplemental Table 2: References for Table 3**

**Supplemental Table 3: References for Table 4**
