# Supplementary material for: Meta-analysis of probability estimates of worldwide variation of CYP2D6 and CYP2C19
Source: Transl Psychiatry. 2021 Feb 24;11:141. doi: 10.1038/s41398-020-01129-1 (PMC7904867; doi:10.1038/s41398-020-01129-1)
Supplement: Supplementary file 2 — Supplemental Table 1: References for Table 2 [file 41398_2020_1129_MOESM2_ESM.doc]

**Supplemental Table 1: References for Table 2**

| Major Geographical Regions | References |
| --- | --- |
| Africa | 1,2,11–20,3,21–29,4–10 |
| African Americans | 27,30,39,40,31–38 |
| Americas | 2,11,45–54,27,55–64,29,65–74,32,75–84,40,85,41–44 |
| Central/ South East Asia | 1,2,88–97,11,98–107,27,108–117,29,118,119,32,34,39,86,87 |
| East Asia | 5,11,61,89,92,98,120–125,18,126–135,27,136–145,29,146–155,32,156–165,34,166–175,39,176–185,40,186,52 |
| Europe | 1,2,29,30,32,34–37,39,40,66,5,103,125,131,187–193,11,194–203,12,204–213,13,214–223,18,224–233,21,234–243,22,244–253,27,254–261 |
| Middle East | 11,34,269–278,39,279–288,262,289–297,263–268 |
| Oceania | 11,40,128,298–300 |

1 Dodgen TM *et al.* Introduction of the AmpliChip CYP450 Test to a South African cohort: a platform comparative prospective cohort study. *BMC Med Genet*; **14**: 20 (2013).

2 Riccardi LN *et al.* CYP2D6 Genotyping in Natives and Immigrants from the Emilia-Romagna Region (Italy). *Genet Test Mol Biomarkers*; **15**: 801–806 (2011).

3 Babalola CP *et al.* Cytochrome P450 CYP2C19 genotypes in Nigerian sickle-cell disease patients and normal controls. *Pharmacogenetics*; **35**: 471–477 (2010).

4 Drögemöller BI *et al.* Characterization of the genetic profile of CYP2C19 in two South African populations. *Pharmacogenomics*; **11**: 1095–1103 (2010).

5 Man M *et al.* Genetic Variation in Metabolizing Enzyme and Transporter Genes: Comprehensive Assessment in 3 Major East Asian Subpopulations With Comparison to Caucasians and Africans. *J Clin Pharmacol*; **50**: 929–940 (2010).

6 Wright GEB *et al.* Elucidation of CYP2D6 Genetic Diversity in a Unique African Population: Implications for the Future Application of Pharmacogenetics in the Xhosa Population. *Ann Hum Genet*; **74**: 340–350 (2010).

7 Kudzi W, Dodoo AN, Mills JJ. Characterisation of CYP2C8, CYP2C9 and CYP2C19 polymorphisms in a Ghanaian population. *BMC Med Genet*; **10**: 124 (2009).

8 Miura J, Obua C, Abbo C, Kaneko S, Tateishi T. Cytochrome P450 2C19 genetic polymorphisms in Ugandans. *Eur J Clin Pharmacol*; **65**: 319–320 (2009).

9 Yen-Revollo JL *et al.* Influence of ethnicity on pharmacogenetic variation in the Ghanaian population. *Pharmacogenomics J*; **9**: 373–379 (2009).

10 Gaedigk A, Coetsee C. The CYP2D6 gene locus in South African Coloureds: unique allele distributions, novel alleles and gene arrangements. *Eur J Clin Pharmacol*; **64**: 465–475 (2008).

11 Sistonen J *et al.* CYP2D6 worldwide genetic variation shows high frequency of altered activity variants and no continental structure. *Pharmacogenet Genomics*; **17**: 93–101 (2007).

12 Allabi AC, Gala J-L, Desager J-P, Heusterspreute M, Horsmans Y. Genetic polymorphisms of CYP2C9 and CYP2C19 in the Beninese and Belgian populations. *Br J Clin Pharmacol*; **56**: 653–657 (2003).

13 Aklillu E, Herrlin K, Gustafsson LL, Bertilsson L, Ingelman-Sundberg M. Evidence for environmental influence on CYP2D6-catalysed debrisoquine hydroxylation as demonstrated by phenotyping and genotyping of Ethiopians living in Ethiopia or in Sweden. *Pharmacogenetics*; **12**: 375–383 (2002).

14 Hamdy SI *et al.* Allele and genotype frequencies of polymorphic cytochromes P450 (CYP2C9, CYP2C19, CYP2E1) and dihydropyrimidine dehydrogenase ( DPYD ) in the Egyptian population. *Br J Clin Pharmacol*; **53**: 596–603 (2002).

15 Bathum L *et al.* Phenotypes and genotypes for CYP2D6 and CYP2C19 in a black Tanzanian population. *Br J Clin Pharmacol*; **48**: 395–401 (1999).

16 Griese E-U, Asante-Poku S, Ofori-Adjei D, Mikus G, Eichelbaum M. Analysis of the CYP2d6 gene mutations and their consequences for enzyme function in a West-African population. *Pharmacogenetics*; **9**: 715–723 (1999).

17 Wennerholm A *et al.* Decreased capacity for debrisoquine metabolism among black Tanzanians. *Pharmacogenetics*; **9**: 707–714 (1999).

18 Droll K *et al.* Comparision of three CYP2D6 probe substrates and genotype in Ghanaians, Chinese and Caucasians. *Pharmacogenetics*; **8**: 325–333 (1998).

19 Persson I, Aklillu E, Rodrigues F, Bertilsson L, Ingelman-Sundberg M. S-mephenytoin hydroxylation phenotype and CYP2C19 genotype among Ethiopians. *Pharmacogenetics*; **6**: 521–526 (1996).

20 Masimirembwa C, Bertilsson L, Johansson I, Hasler JA, Ingelman-Sundberg M. Phenotyping and genotyping of S-mephenytoin hydroxylase (cytochrome P450 2C19) in a Shona population of Zimbabwe. *Clin Pharmacol Ther*; **57**: 656–661 (1995).

21 Evans WE *et al.* Genetic basis for a lower prevalence of deficient CYP2D6 oxidative drug metabolism phenotypes in black Americans. *J Clin Invest*; **91**: 2150–2154 (1993).

22 Riccardi LN *et al.* CYP2D6 polymorphism studies: How forensic genetics helps clinical medicine. *Forensic Sci Int Genet Suppl Ser*; **2**: 485–486 (2009).

23 Wennerholm A *et al.* Characterization of the CYP2D6*29 allele commonly present in a black Tanzanian population causing reduced catalytic activity. *Pharmacogenetics*; **11**: 417–27 (2001).

24 Herrlin K *et al.* Bantu Tanzanians have a decreased capacity to metabolize omeprazole and mephenytoin in relation to their CYP2C19 genotype*. *Clin Pharmacol Ther*; **64**: 391–401 (1998).

25 Masimirembwa C *et al.* Phenotype and genotype analysis of debrisoquine hydroxylase (CYP2D6) in a black Zimbabwean population. *Eur J Clin Pharmacol*; **51**: 117–122 (1996).

26 Afilal D *et al.* Genetic Polymorphism of Drug-Metabolizing Enzymes CYP2C9 and CYP2C19 in Moroccan Population. *Genet Test Mol Biomarkers*; **21**: 298–304 (2017).

27 Wendt FR, Pathak G, Sajantila A, Chakraborty R, Budowle B. Global genetic variation of select opiate metabolism genes in self-reported healthy individuals. *Pharmacogenomics J*; **18**: 281–294 (2018).

28 Dodgen TM *et al.* Pharmacogenetic comparison of CYP2D6 predictive and measured phenotypes in a South African cohort. *Pharmacogenomics J*; **16**: 566–572 (2016).

29 Zhou Y *et al.* Worldwide Distribution of Cytochrome P450 Alleles: A Meta-analysis of Population-scale Sequencing Projects. *Genet Med*; **21**: 1345–1354 (2019).

30 Martis S *et al.* Multi-ethnic distribution of clinically relevant CYP2C genotypes and haplotypes. *Pharmacogenomics J*; **13**: 369–377 (2013).

31 Cai W-M *et al.* CYP2D6 genetic variation in healthy adults and psychiatric African-American subjects: implications for clinical practice and genetic testing. *Pharmacogenomics J*; **6**: 343–350 (2006).

32 Luo H, Poland R, Lin K, Wan Y. Genetic polymorphism of cytochrome P450 2C19 in Mexican Americans: A cross-ethnic comparative study. *Clin Pharmacol Ther*; **80**: 33–40 (2006).

33 Gaedigk A *et al.* Identification and characterization of novel sequence variations in the cytochrome P4502D6 (CYP2D6) gene in African Americans. *Pharmacogenomics J*; **5**: 173–182 (2005).

34 Melis R, Lyon E, McMillin GA. Determination of CYP2D6 , CYP2C9 and CYP2C19 genotypes with Tag–It ^TM^ mutation detection assays. *Expert Rev Mol Diagn*; **6**: 811–820 (2006).

35 Gaedigk A, Bradford LD, Marcucci K a, Leeder JS. Unique CYP2D6 activity distribution and genotype-phenotype discordance in black Americans. *Clin Pharmacol Ther*; **72**: 76–89 (2002).

36 Wan Y-JY *et al.* Analysis of the CYP2D6 gene polymorphism and enzyme activity in African-Americans in Southern California. *Pharmacogenetics*; **11**: 489–499 (2001).

37 Leathart JB *et al.* CYP2D6 phenotype-genotype relationships in African-Americans and Caucasians in Los Angeles. *Pharmacogenetics*; **8**: 529–41 (1998).

38 Marinac JS *et al.* Determination of CYP2C19 phenotype in black Americans with omeprazole: Correlation with genotype. *Clin Pharmacol Ther*; **60**: 138–144 (1996).

39 Goldstein JA *et al.* Frequencies of the defective CYP2C19 alleles responsible for the mephenytoin poor metabolizer phenotype in various Oriental, Caucasian, Saudi Arabian and American black populations. *Pharmacogenetics*; **7**: 59–64 (1997).

40 Budd WT *et al.* Next generation sequencing reveals disparate population frequencies among cytochrome P450 genes: clinical pharmacogenomics of the CYP2 family. *Int J Comput Biol Drug Des*; **9**: 54 (2016).

41 Kohlrausch FB, Carracedo Á, Hutz MH. Characterization of CYP1A2, CYP2C19, CYP3A4 and CYP3A5 polymorphisms in South Brazilians. *Mol Biol Rep*; **41**: 1453–1460 (2014).

42 Lazalde-Ramos BP *et al.* CYP2D6 gene polymorphisms and predicted phenotypes in eight indigenous groups from northwestern Mexico. *Pharmacogenomics*; **15**: 339–348 (2014).

43 Fohner A *et al.* Pharmacogenetics in American Indian populations. *Pharmacogenet Genomics*; **23**: 403–414 (2013).

44 Montané Jaime LK, Lalla A, Steimer W, Gaedigk A. Characterization of the CYP2D6 gene locus and metabolic activity in Indo- and Afro-Trinidadians: discovery of novel allelic variants. *Pharmacogenomics*; **14**: 261–276 (2013).

45 Griman P *et al.* CYP2D6 gene variants in urban/admixed and Amerindian populations of Venezuela: Pharmacogenetics and anthropological implications. *Ann Hum Biol*; **39**: 137–142 (2012).

46 Llerena A *et al.* CYP2D6 genotype and debrisoquine hydroxylation phenotype in Cubans and Nicaraguans. *Pharmacogenomics J*; **12**: 176–83 (2012).

47 Suarez-Kurtz G *et al.* Global pharmacogenomics: Impact of population diversity on the distribution of polymorphisms in the CYP2C cluster among Brazilians. *Pharmacogenomics J*; **12**: 267–276 (2012).

48 Contreras A V *et al.* Resequencing, haplotype construction and identification of novel variants of CYP2D6 in Mexican Mestizos. *Pharmacogenomics*; **12**: 745–756 (2011).

49 Sosa-Macías M, Dorado P, Alanis-Bañuelos RE, Llerena A, Lares-Asseff I. Influence of CYP2D6 Deletion, Multiplication, –1584C→G, 31G→A and 2988G→A Gene Polymorphisms on Dextromethorphan Metabolism among Mexican Tepehuanos and Mestizos. *Pharmacology*; **86**: 30–36 (2010).

50 Kohlrausch FB *et al.* Molecular diversity at the CYP2D6 locus in healthy and schizophrenic southern Brazilians. *Pharmacogenomics*; **10**: 1457–1466 (2009).

51 Maciel ME *et al.* Population analysis of xenobiotic metabolizing genes in South Brazilian Euro and Afro-descendants. *Genet Mol Biol*; **32**: 723–728 (2009).

52 Perini JA *et al.* Pharmacogenetic polymorphisms in Brazilian-born, first-generation Japanese descendants. *Brazilian J Med Biol Res*; **42**: 1179–84 (2009).

53 Silveira VDS, Canalle R, Scrideli CA, Queiroz RGDP, Tone LG. Polymorphisms in genes encoding drugs and xenobiotic metabolizing enzymes in a Brazilian population. *Biomarkers*; **14**: 111–117 (2009).

54 Bailliet G *et al.* Allele and genotype frequencies of metabolic genes in Native Americans from Argentina and Paraguay. *Mutat Res Toxicol Environ Mutagen*; **627**: 171–177 (2007).

55 Isaza C, Henao J, Martínez JHI, Arias JCS, Beltrán L. Phenotype-genotype analysis of CYP2C19 in Colombian mestizo individuals. *BMC Clin Pharmacol*; **7**: 6 (2007).

56 Bravo-Villalta H V. *et al.* Genetic polymorphism of CYP2C9 and CYP2C19 in a Bolivian population: an investigative and comparative study. *Eur J Clin Pharmacol*; **61**: 179–184 (2005).

57 Luo H-R, Gaedigk A, Aloumanis V, Wan Y-JY. Identification of CYP2D6 impaired functional alleles in Mexican Americans. *Eur J Clin Pharmacol*; **61**: 797–802 (2005).

58 Isaza CA, Henao J, López AM, Cacabelos R. Isolation, sequence and genotyping of the drug metabolizer CYP2D6 gene in the Colombian population. *Methods Find Exp Clin Pharmacol*; **22**: 695–705 (2000).

59 Jorge LF, Eichelbaum M, Griese EU, Inaba T, Arias TD. Comparative evolutionary pharmacogenetics of CYP2D6 in Ngawbe and Embera Amerindians of Panama and Colombia: role of selection versus drift in world populations. *Pharmacogenetics*; **9**: 217–28 (1999).

60 Muñoz S *et al.* Genetic polymorphisms of CYP2D6, CYP1A1 and CYP2E1 in the South-Amerindian population of Chile. *Pharmacogenetics*; **8**: 343–51 (1998).

61 Nowak MP, Sellers EM, Tyndale RF. Canadian Native Indians exhibit unique CYP2A6 and CYP2C19 mutant allele frequencies*. *Clin Pharmacol Ther*; **64**: 378–383 (1998).

62 Agúndez JA, Ramirez R, Hernandez M, Llerena A, Benítez J. Molecular heterogeneity at the CYP2D gene locus in Nicaraguans: impact of gene-flow from Europe. *Pharmacogenetics*; **7**: 337–40 (1997).

63 Jurima-Romet M *et al.* CYP2D6-related oxidation polymorphism in a Canadian Inuit population. *Can J Physiol Pharmacol*; **75**: 165–172 (1997).

64 Nowak MP, Tyndale RF, Sellers EM. CYP2D6 phenotype and genotype in a Canadian Native Indian population. *Pharmacogenetics*; **7**: 145–8 (1997).

65 Jurima-Romet M *et al.* CYP2C19 genotyping and associated mephenytoin hydroxylation polymorphism in a Canadian Inuit population. *Pharmacogenetics*; **6**: 329–39 (1996).

66 Vicente J *et al.* Genetic polymorphisms of CYP2C8, CYP2C9 and CYP2C19 in Ecuadorian Mestizo and Spaniard populations: a comparative study. *Mol Biol Rep*; **41**: 1267–1272 (2014).

67 Dorado P *et al.* CYP2D6 genotype and dextromethorphan hydroxylation phenotype in an Ecuadorian population. *Eur J Clin Pharmacol*; **68**: 637–644 (2012).

68 Salazar-Flores J *et al.* Distribution of CYP2D6 and CYP2C19 Polymorphisms Associated with Poor Metabolizer Phenotype in Five Amerindian Groups and Western Mestizos from Mexico. *Genet Test Mol Biomarkers*; **16**: 1098–1104 (2012).

69 Vargens DD, Petzl-Erler M-L, Suarez-Kurtz G. Distribution of CYP2C Polymorphisms in an Amerindian Population of Brazil. *Basic Clin Pharmacol Toxicol*; **110**: 396–400 (2012).

70 Chiurillo MA, Grimán P, Morán Y, Camargo ME, Ramírez JL. Analysis of CYP2D6 gene variation in Venezuelan population: Implications for forensic toxicology. *Forensic Sci Int Genet Suppl Ser*; **2**: 483–484 (2009).

71 López M, Guerrero J, Jung–Cook H, Alonso ME. CYP2D6 genotype and phenotype determination in a Mexican Mestizo population. *Eur J Clin Pharmacol*; **61**: 749–754 (2005).

72 de Andrés F, Sosa-Macías M, Ramos BPL, Naranjo M-EG, LLerena A. CYP450 Genotype/Phenotype Concordance in Mexican Amerindian Indigenous Populations–Where to from Here for Global Precision Medicine? *Omi A J Integr Biol*; **21**: 509–519 (2017).

73 Koopmans AB, Vinkers DJ, Gelan PJ, Hoek HW, van Harten PN. CYP2D6 and CYP2C19 genotyping in psychiatric patients on psychotropic medication in the former Dutch Antilles. *Pharmacogenomics*; **18**: 1003–1012 (2017).

74 Acuña M, Pinto E, Olivares P, Ríos C. Genetic Variants of Cytochrome CYP2D6 in Two Mixed Chilean Populations. *Hum Hered*; **82**: 16–20 (2016).

75 Céspedes-Garro C *et al.* Relevance of the ancestry for the variability of the Drug-Metabolizing Enzymes CYP2C9, CYP2C19 and CYP2D6 polymorphisms in a multiethnic Costa Rican population. *Rev Biol Trop*; **64** (2016). doi:10.15517/rbt.v64i3.20901.

76 de Andrés F, Terán S, Hernández F, Terán E, LLerena A. To genotype or phenotype for personalized medicine? CYP450 drug metabolizing enzyme genotype–phenotype concordance and discordance in the Ecuadorian population. *Omi A J Integr Biol*; **20**: 699–710 (2016).

77 Ortega-Vázquez A *et al.* CYP2C9, CYP2C19, ABCB1 genetic polymorphisms and phenytoin plasma concentrations in Mexican-Mestizo patients with epilepsy. *Pharmacogenomics J*; **16**: 286–292 (2016).

78 Saldaña-Cruz AM *et al.* CYP2C9 and CYP2C19 Allele and Haplotype Distributions in Four Mestizo Populations from Western Mexico: An Interethnic Comparative Study. *Genet Test Mol Biomarkers*; **20**: 702–709 (2016).

79 Varela N *et al.* Characterization of the CYP2D6 drug metabolizing phenotypes of the Chilean mestizo population through polymorphism analyses. *Pharmacol Res*; **101**: 124–129 (2015).

80 Friedrich DC *et al.* Distribution of CYP2D6 Alleles and Phenotypes in the Brazilian Population. *PLoS One*; **9**: e110691 (2014).

81 López-López M *et al.* CYP2D6 genetic polymorphisms in Southern Mexican Mayan Lacandones and Mestizos from Chiapas. *Pharmacogenomics*; **15**: 1859–1865 (2014).

82 Espinoza N, Galdames J, Navea D, Farfán MJ, Salas C. Frequency of the CYP2C19*17 polymorphism in a Chilean population and its effect on voriconazole plasma concentration in immunocompromised children. *Sci Rep*; **9**: 8863 (2019).

83 León-Moreno LC *et al.* Distribution of potential risk alleles and haplotypes of the CYP2C9 and CYP2C19 genes in Mexican native populations: A comparative study among Amerindian populations. *Meta Gene*; **20**: 100565 (2019).

84 Favela‐Mendoza AF *et al.* Correspondence between the CYP2C19 and CYP3A4 genotypes with the inferred metabolizer phenotype by omeprazole administration in Mexican healthy children. *J Clin Pharm Ther*; **43**: 656–663 (2018).

85 Naranjo M-EG *et al.* Interethnic Variability in CYP2D6 , CYP2C9 , and CYP2C19 Genes and Predicted Drug Metabolism Phenotypes Among 6060 Ibero- and Native Americans: RIBEF-CEIBA Consortium Report on Population Pharmacogenomics. *Omi A J Integr Biol*; **22**: 575–588 (2018).

86 Shalia KK, Shah VK, Pawar P, Divekar SS, Payannavar S. Polymorphisms of MDR1, CYP2C19 and P2Y12 genes in Indian population: Effects on clopidogrel response. *Indian Heart J*; **65**: 158–167 (2013).

87 Anichavezhi D, Chakradhara Rao US, Shewade DG, Krishnamoorthy R, Adithan C. Distribution of CYP2C19*17 allele and genotypes in an Indian population. *J Clin Pharm Ther*; **37**: 313–318 (2012).

88 Suwannasri P, Thongnoppakhun W, Pramyothin P, Assawamakin A, Limwongse C. Combination of multiplex PCR and DHPLC-based strategy for CYP2D6 genotyping scheme in Thais. *Clin Biochem*; **44**: 1144–1152 (2011).

89 Kim E *et al.* Robust CYP2D6 genotype assay including copy number variation using multiplex single-base extension for Asian populations. *Clin Chim Acta*; **411**: 2043–2048 (2010).

90 Veiga MI *et al.* Pharmacogenomics of CYP2A6, CYP2B6, CYP2C19, CYP2D6, CYP3A4, CYP3A5 and MDR1 in Vietnam. *Eur J Clin Pharmacol*; **65**: 355–363 (2009).

91 Ghodke Y *et al.* Genetic polymorphism of CYP2C19 in Maharashtrian population. *Eur J Epidemiol*; **22**: 907–915 (2007).

92 Lee SS *et al.* Comparisons of CYP2C19 Genetic Polymorphisms Between Korean and Vietnamese Populations. *Ther Drug Monit*; **29**: 455–459 (2007).

93 Tassaneeyakul W *et al.* CYP2C19 Genetic Polymorphism in Thai, Burmese and Karen Populations. *Drug Metab Pharmacokinet*; **21**: 286–290 (2006).

94 Jose R *et al.* CYP2C9 and CYP2C19 genetic polymorphisms: frequencies in the south Indian population. *Fundam Clin Pharmacol*; **19**: 101–105 (2005).

95 Yang YS *et al.* Genetic polymorphism of cytochrome P450 2C19 in healthy Malaysian subjects. *Br J Clin Pharmacol*; **58**: 332–335 (2004).

96 Adithan C *et al.* Allele and genotype frequency of CYP2C19 in a Tamilian population. *Br J Clin Pharmacol*; **56**: 331–333 (2003).

97 Tassaneeyakul W *et al.* Analysis of the CYP2C19 polymorphism in a North-eastern Thai population. *Pharmacogenetics*; **12**: 221–225 (2002).

98 Ismail R, Teh LK, Amir J, Alwi Z, Lopez CG. Genetic polymorphism of CYP2D6 in Chinese subjects in Malaysia. *J Clin Pharm Ther*; **28**: 279–84 (2003).

99 Teh LK *et al.* Heterogeneity of the CYP2D6 gene among Malays in Malaysia. *J Clin Pharm Ther*; **26**: 205–211 (2001).

100 Gulati S *et al.* Frequency distribution of high risk alleles of CYP2C19, CYP2E1, CYP3A4 genes in Haryana population. *Environ Toxicol Pharmacol*; **37**: 1186–1193 (2014).

101 Baclig MO *et al.* Allelic and genotype frequencies of catechol-O-methyltransferase (Val158Met) and CYP2D6*10 (Pro34Ser) single nucleotide polymorphisms in the Philippines. *Int J Mol Epidemiol Genet*; **3**: 115–21 (2012).

102 Siddapuram SP *et al.* CYP2C19 polymorphism as a predictor of personalized therapy in South Indian population. *J Assoc Physicians India*; **59**: 490–3 (2011).

103 Sistonen J *et al.* Pharmacogenetic variation at CYP2C9, CYP2C19, and CYP2D6 at global and microgeographic scales. *Pharmacogenet Genomics*; **19**: 170–179 (2009).

104 Majumdar S *et al.* Association of cytochrome P450, glutathione S-transferase and N-acetyl transferase 2 gene polymorphisms with incidence of acute myeloid leukemia. *Eur J Cancer Prev*; **17**: 125–132 (2008).

105 Singh M *et al.* Polymorphism in environment responsive genes and association with Parkinson disease. *Mol Cell Biochem*; **312**: 131–138 (2008).

106 Naveen AT, Adithan C, Soya SS, Gerard N, Krishnamoorthy R. CYP2D6 Genetic Polymorphism in South Indian Populations. *Biol Pharm Bull*; **29**: 1655–1658 (2006).

107 Buch S, Kotekar A, Kawle D, Bhisey R. Polymorphisms at CYP and GST gene loci. Prevalence in the Indian population. *Eur J Clin Pharmacol*; **57**: 553–5 (2001).

108 Weerasuriya K *et al.* Debrisoquine and mephenytoin oxidation in Sinhalese: a population study. *Br J Clin Pharmacol*; **38**: 466–470 (1994).

109 Deshpande N *et al.* Rapid and ultra-rapid metabolizers with CYP2C19 *17 polymorphism do not respond to standard therapy with proton pump inhibitors. *Meta Gene*; **9**: 159–164 (2016).

110 Sridharan K *et al.* Evaluation of CYP2C19, P2Y12, and ABCB1 polymorphisms and phenotypic response to clopidogrel in healthy Indian adults. *Indian J Pharmacol*; **48**: 350 (2016).

111 Ayyappadhas R, Dhanalekshmi U, Jestin C. CYP 2D6*4 polymorphism and interindividual response variation to metoprolol instage 1 hypertensive patients: no association in a rural Indian population? *Turkish J Med Sci*; **45**: 352–357 (2015).

112 Choudhury S, Akam E, Mastana S. Genetic Analysis of CYP2D6 Polymorphism in Indian Populations and its Pharmacogenetic Implications. *Curr Pharmacogenomics Person Med*; **12**: 123–132 (2014).

113 Afsar NA *et al.* Implications of genetic variation of common Drug Metabolizing Enzymes and ABC Transporters among the Pakistani Population. *Sci Rep*; **9**: 7323 (2019).

114 Nguyen HH *et al.* Single nucleotide and structural variants of CYP2D6 gene in Kinh Vietnamese population. *Medicine (Baltimore)*; **98**: e15891 (2019).

115 Riaz S *et al.* Genetic Polymorphism of CYP2C19 in Pakistani Population. *Iran J Pharm Res*; **18**: 1097–1102 (2019).

116 Bhat MA, Gandhi G. CYP2D6 (C2850T, G1846A, C100T) polymorphisms, haplotypes and MDR analysis in predicting coronary artery disease risk in north-west Indian population: A case-control study. *Gene*; **663**: 17–24 (2018).

117 Paradkar MU, Shah SAV, Dherai AJ, Shetty D, Ashavaid TF. Distribution of CYP2D6 genotypes in the Indian population – preliminary report. *Drug Metab Pers Ther*; **33**: 141–151 (2018).

118 Riaz S *et al.* Association of CYP2C19 * 2 and * 17 genetic variants with hypertension in Pakistani population. *Trop J Pharm Res*; **18**: 851–855 (2019).

119 Singh H, Lata S, Gangakhedkar RR. Prevalence of CYP2D6 * 4 1934G / A polymorphism in Western Indian HIV patients. *J Pathol Microbiol Immunol*; **126**: 842–851 (2018).

120 Xiuchun Y, Fan L, Jingchao L, Bing X, Wei C. GW24-e1197 Allele and genotype frequencies of CYP2C19 in Chinese Han population. *Heart*; **99**: e128 (2013).

121 Jin T *et al.* Polymorphisms and phenotypic analysis of cytochrome P450 2D6 in the Tibetan population. *Gene*; **527**: 360–365 (2013).

122 Shin D-J *et al.* Association of CYP2C19*2 and *3 Genetic Variants with Essential Hypertension in Koreans. *Yonsei Med J*; **53**: 1113 (2012).

123 Yin S-J *et al.* Differences in genotype and allele frequency distributions of polymorphic drug metabolizing enzymes CYP2C19 and CYP2D6 in mainland Chinese Mongolian, Hui and Han populations. *J Clin Pharm Ther*; **37**: 364–369 (2012).

124 Zuo J, Xia D, Jia L, Guo T. Genetic polymorphisms of drug-metabolizing phase I enzymes CYP3A4, CYP2C9, CYP2C19 and CYP2D6 in Han, Uighur, Hui and Mongolian Chinese populations. *Pharmazie*; **67**: 639–644 (2011).

125 Ramsjö M *et al.* CYP2C19 activity comparison between Swedes and Koreans: effect of genotype, sex, oral contraceptive use, and smoking. *Eur J Clin Pharmacol*; **66**: 871–877 (2010).

126 Kim KA, Song WK, Park JY. Association of CYP2B6, CYP3A5, and CYP2C19 Genetic Polymorphisms With Sibutramine Pharmacokinetics in Healthy Korean Subjects. *Clin Pharmacol Ther*; **86**: 511–518 (2009).

127 Lee S-J *et al.* Discovery of Novel Functional Variants and Extensive Evaluation of CYP2D6 Genetic Polymorphisms in Koreans. *Drug Metab Dispos*; **37**: 1464–1470 (2009).

128 Wang S-M *et al.* Frequencies of genotypes and alleles of the functional SNPs in CYP2C19 and CYP2E1 in mainland Chinese Kazakh, Uygur and Han populations. *J Hum Genet*; **54**: 372–375 (2009).

129 Zhou Q *et al.* Genetic polymorphism, linkage disequilibrium, haplotype structure and novel allele analysis of CYP2C19 and CYP2D6 in Han Chinese. *Pharmacogenomics J*; **9**: 380–394 (2009).

130 Chen L *et al.* Genetic polymorphism analysis of CYP2C19 in Chinese Han populations from different geographic areas of mainland China. *Pharmacogenomics*; **9**: 691–702 (2008).

131 Myrand S *et al.* Pharmacokinetics/Genotype Associations for Major Cytochrome P450 Enzymes in Native and First- and Third-generation Japanese Populations: Comparison With Korean, Chinese, and Caucasian Populations. *Clin Pharmacol Ther*; **84**: 347–361 (2008).

132 Qin S *et al.* Systematic polymorphism analysis of the CYP2D6 gene in four different geographical Han populations in mainland China. *Genomics*; **92**: 152–158 (2008).

133 Sugimoto K, Uno T, Yamazaki H, Tateishi T. Limited frequency of the CYP2C19*17 allele and its minor role in a Japanese population. *Br J Clin Pharmacol*; **65**: 437–439 (2008).

134 Cai WM, Chen B, Zhang WX. Frequency of CYP2D6*10 and *14 Alleles and their Influence on the Metabolic Activity of CYP2D6 in a Healthy Chinese Population. *Clin Pharmacol Ther*; **81**: 95–98 (2007).

135 Ishii G, Suzuki A, Oshino S, Shiraishi H, Otani K. CYP2C19 polymorphism affects personality traits of Japanese females. *Neurosci Lett*; **411**: 77–80 (2007).

136 Iwashima K *et al.* No association between CYP2D6 polymorphisms and personality trait in Japanese. *Br J Clin Pharmacol*; **64**: 96–99 (2007).

137 Sheng H-H *et al.* Allelic distributions of CYP2D6 gene copy number variation in the Eastern Han Chinese population. *Acta Pharmacol Sin*; **28**: 279–286 (2007).

138 Lee S-Y *et al.* Sequence-based CYP2D6 Genotyping in the Korean Population. *Ther Drug Monit*; **28**: 382–387 (2006).

139 Liou Y-H, Lin C-T, Wu Y-J, Wu LS-H. The high prevalence of the poor and ultrarapid metabolite alleles of CYP2D6, CYP2C9, CYP2C19, CYP3A4, and CYP3A5 in Taiwanese population. *J Hum Genet*; **51**: 857–863 (2006).

140 Fukushima-Uesaka H *et al.* Genetic variations and haplotypes of CYP2C19 in a Japanese population. *Drug Metab Pharmacokinet*; **20**: 300–7 (2005).

141 Ikenaga Y *et al.* The Frequency of Candidate Alleles for CYP2D6 Genotyping in the Japanese Population with an Additional Respect to the −1584C to G Substitution. *Drug Metab Pharmacokinet*; **20**: 113–116 (2005).

142 Ji L *et al.* Single-step assays to analyze CYP2D6 gene polymorphisms in Asians: allele frequencies and a novel *14B allele in mainland Chinese. *Clin Chem*; **48**: 983–8 (2002).

143 Mitsunaga Y *et al.* Frequent occurrence of CYP2D6∗10 duplication allele in a Japanese population. *Mutat Res Mol Mech Mutagen*; **505**: 83–85 (2002).

144 Kimura M, Ieiri I, Mamiya K, Urae A, Higuchi S. Genetic polymorphism of cytochrome P450s, CYP2C19, and CYP2C9 in a Japanese population. *Ther Drug Monit*; **20**: 243–7 (1998).

145 Xiao ZS *et al.* Differences in the incidence of the CYP2C19 polymorphism affecting the S-mephenytoin phenotype in Chinese Han and Bai populations and identification of a new rare CYP2C19 mutant allele. *J Pharmacol Exp Ther*; **281**: 604–9 (1997).

146 Kubota T, Chiba K, Ishizaki T. Genotyping of S-mephenytoin 4’-hydroxylation in an extended Japanese population. *Clin Pharmacol Ther*; **60**: 661–6 (1996).

147 Roh HK *et al.* CYP2C19 genotype and phenotype determined by omeprazole in a Korean population. *Pharmacogenetics*; **6**: 547–51 (1996).

148 Roh HK *et al.* Debrisoquine and S-mephenytoin hydroxylation phenotypes and genotypes in a Korean population. *Pharmacogenetics*; **6**: 441–7 (1996).

149 Li J *et al.* Correlation between CYP2D6*10 Gene Mutation, and Structure and Function of its Encoding Prot. *Trop J Pharm Res*; **13**: 347 (2014).

150 Yan F, Xu J-F, Liu X, Li X-H. Interaction between smoking and CYP2C19*3 polymorphism increased risk of lung cancer in a Chinese population. *Tumor Biol*; **35**: 5295–5298 (2014).

151 Chan SW *et al.* CYP2C19 genotype has a major influence on labetalol pharmacokinetics in healthy male Chinese subjects. *Eur J Clin Pharmacol*; **69**: 799–806 (2013).

152 Qian J-C *et al.* Genetic variations of human CYP2D6 in the Chinese Han population. *Pharmacogenomics*; **14**: 1731–1743 (2013).

153 Wu Z *et al.* A Systematically Combined Genotype and Functional Combination Analysis of CYP2E1, CYP2D6, CYP2C9, CYP2C19 in Different Geographic Areas of Mainland China - A Basis for Personalized Therapy. *PLoS One*; **8** (2013). doi:10.1371/journal.pone.0071934.

154 Hu L-M *et al.* Genetic polymorphisms and novel allelic variants of CYP2C19 in the Chinese Han population. *Pharmacogenomics*; **13**: 1571–1581 (2012).

155 Okubo M, Murayama N, Miura J, Shimizu M, Yamazaki H. A rapid multiplex PCR assay that can reliably discriminate the cytochrome P450 2D6 whole-gene deletion allele from 2D6*10 alleles. *Clin Chim Acta*; **413**: 1675–1677 (2012).

156 Shao H *et al.* Influence of CYP2C9 and CYP2C19 genetic polymorphisms on pharmacokinetics and pharmacodynamics of gliclazide in healthy Chinese Han volunteers. *J Clin Pharm Ther*; **35**: 351–360 (2010).

157 Yang Y-N *et al.* Association of Interaction Between Smoking and CYP 2C19*3 Polymorphism With Coronary Artery Disease in a Uighur Population. *Clin Appl Thromb*; **16**: 579–583 (2010).

158 Yang ZF *et al.* Genetic polymorphisms of cytochrome P450 enzymes 2C9 and 2C19 in a healthy Mongolian population in China. *Genet Mol Res*; **9**: 1844–1851 (2010).

159 Yoo HD, Park SA, Cho HY, Lee YB. Influence of CYP3A and CYP2C19 genetic polymorphisms on the pharmacokinetics of cilostazol in healthy subjects. *Clin Pharmacol Ther*; **86**: 281–284 (2009).

160 Otani K *et al.* Reduced CYP2D6 activity is a negative risk factor for methamphetamine dependence. *Neurosci Lett*; **434**: 88–92 (2008).

161 Kato D *et al.* Effects of CYP2D6 polymorphisms on neuroleptic malignant syndrome. *Eur J Clin Pharmacol*; **63**: 991–996 (2007).

162 Wang J-H, Li P-Q, Fu Q-Y, Li Q-X, Cai W-W. CYP2C19 genotype and omeprazole hydroxylation phenotype in Chinese Li population. *Clin Exp Pharmacol Physiol*; **34**: 421–424 (2007).

163 Kim M-K *et al.* Effect of the CYP2D6 genotype on the pharmacokinetics of tropisetron in healthy Korean subjects. *Eur J Clin Pharmacol*; **59**: 111–116 (2003).

164 Ling J, Shixiu P, Jianmin W, Marti-Jaun J, Hersberger M. Genetic polymorphism of CYP2D6 in Chinese mainland. *Chin Med J (Engl)*; **115**: 1780–1784 (2002).

165 Ohkubo T, Suno M, Sugawara K, Motomuro S. Graphic Roots of CYP2C19 genetic polymorphism in Japanese population (preliminary report). *Int Congr Ser*; **1244**: 63–67 (2002).

166 Tateishi T. Analysis of the CYP2D6 gene in relation to its metabolic capacity in Japanese men and in comparison with other populations. *Int Congr Ser*; **1244**: 33–39 (2002).

167 Chida M *et al.* A new variant CYP2D6 allele (CYP2D6*21) with a single base insertion in exon 5 in a Japanese population associated with a poor metabolizer phenotype. *Pharmacogenetics*; **9**: 287–93 (1999).

168 Tateishi T *et al.* Analysis of the gene in relation to dextromethorphan -demethylation capacity in a Japanese population. *Clin Pharmacol Ther*; **65**: 570–575 (1999).

169 Takakubo F, Kuwano A, Kondo I. Evidence that poor metabolizers of (S)-mephenytoin could be identified by haplotypes of CYP2C19 in Japanese. *Pharmacogenetics*; **6**: 265–267 (1996).

170 Tsuneoka Y, Fukushima K, Matsuo Y, Ichikawa Y, Watanabe Y. Genotype analysis of the CYP2C19 gene in the Japanese population. *Life Sci*; **59**: 1711–5 (1996).

171 Akasaka T *et al.* Sex differences in the impact of CYP2C19 polymorphisms and low-grade inflammation on coronary microvascular disorder. *Am J Physiol Circ Physiol*; **310**: H1494–H1500 (2016).

172 Jeon J-Y, Chae S-W, Kim M-G. Population pharmacokinetics of aripiprazole in healthy Korean subjects. *Int J Clin Pharmacol Ther*; **54**: 293–304 (2016).

173 Jin T *et al.* Genotype-phenotype analysis of CYP2C19 in the Tibetan population and its potential clinical implications in drug therapy. *Mol Med Rep*; **13**: 2117–2123 (2016).

174 Chen R, Wang H, Shi J, Shen K, Hu P. Cytochrome P450 2D6 genotype affects the pharmacokinetics of controlled-release paroxetine in healthy Chinese subjects: comparison of traditional phenotype and activity score systems. *Eur J Clin Pharmacol*; **71**: 835–841 (2015).

175 Cho D-Y *et al.* Effect of the potent CYP2D6 inhibitor sarpogrelate on the pharmacokinetics and pharmacodynamics of metoprolol in healthy male Korean volunteers. *Xenobiotica*; **45**: 256–263 (2015).

176 Ding Y *et al.* Genetic polymorphisms and phenotypic analysis of drug-metabolizing enzyme CYP2C19 in a Li Chinese population. *Int J Clin Exp Pathol*; **8**: 13201–8 (2015).

177 Dong Y *et al.* Analysis of genetic variations in CYP2C9, CYP2C19, CYP2D6 and CYP3A5 genes using oligonucleotide microarray. *Int J Clin Exp Med*; **8**: 18917–26 (2015).

178 Hokimoto S *et al.* Gender Differences in Impact of CYP2C19 Polymorphism on Development of Coronary Artery Disease. *J Cardiovasc Pharmacol*; **65**: 1 (2014).

179 Jin T *et al.* Genetic polymorphisms of the drug-metabolizing enzyme CYP2C19 in the Uyghur population in northwest China. *Xenobiotica*; **46**: 634–640 (2016).

180 Moon SJ, Yoon J, Oh J, Lee S, Yu K-S. Population Pharmacokinetics of Voriconazole in Healthy Korean Male with Various CYP2C19 Genotypes. *J Pharmacokinet Pharmacodyn*; **42**: 759 (2015).

181 Li J *et al.* Statin therapy correlated CYP2D6 gene polymorphism and hyperlipidemia. *Curr Med Res Opin*; **30**: 223–228 (2014).

182 Zhong Z *et al.* Analysis of CYP2C19 Genetic Polymorphism in a Large Ethnic Hakka Population in Southern China. *Med Sci Monit*; **23**: 6186–6192 (2017).

183 Lee J, Yoo H-D, Bae J-W, Lee S, Shin K-H. Population pharmacokinetic analysis of tramadol and O-desmethyltramadol with genetic polymorphism of CYP2D6. *Drug Des Devel Ther*; **Volume 13**: 1751–1761 (2019).

184 Byeon J-Y *et al.* CYP2D6 allele frequencies in Korean population, comparison with East Asian, Caucasian and African populations, and the comparison of metabolic activity of CYP2D6 genotypes. *Arch Pharm Res*; **41**: 921–930 (2018).

185 Cao P *et al.* Genetic polymorphisms of the drug-metabolizing enzyme CYP2J2 in a Tibetan population. *Medicine (Baltimore)*; **97**: e12579 (2018).

186 Jung E *et al.* Influence of CYP2D6 Polymorphism on the Pharmacokinetic/Pharmacodynamic Characteristics of Carvedilol in Healthy Korean Volunteers. *J Korean Med Sci*; **33**: 1–12 (2018).

187 Jakovski K, Nestorovska AK, Labacevski N, Dimovski AJ. Characterization of the most common CYP2C9 and CYP2C19 allelic variants in the population from the Republic of Macedonia. *Pharmazie*; **68**: 893–8 (2013).

188 Sipeky C *et al.* High prevalence of CYP2C19*2 allele in Roma samples: study on Roma and Hungarian population samples with review of the literature. *Mol Biol Rep*; **40**: 4727–4735 (2013).

189 Korchagina RP *et al.* Polymorphisms of the GSTM1, GSTT1, and CYP2D6 xenobiotic biotransformation genes, which are possible risk markers of cancer in populations of indigenous ethnic groups and Russians of North Siberia. *Russ J Genet Appl Res*; **2**: 7–17 (2012).

190 Scott SA *et al.* Identification of CYP2C19*4B: Pharmacogenetic implications for drug metabolism including clopidogrel responsiveness. *Pharmacogenomics J*; **12**: 297–305 (2012).

191 Stingl JC *et al.* Genetic variation in CYP2D6 impacts neural activation during cognitive tasks in humans. *Neuroimage*; **59**: 2818–2823 (2012).

192 Buzoianu AD *et al.* Screening for CYP2C19*2, *3 AND *4 gene variants in a Romanian population study group. *Farmacia*; **58**: 806–817 (2010).

193 Fernández-Santander A *et al.* CYP2D6 polymorphism screening in a selected population of Spain (La Alpujarra): No effect of geographical isolation. *Ann Hum Biol*; **37**: 268–274 (2010).

194 Gra O *et al.* Microarray-Based Detection of CYP1A1 , CYP2C9 , CYP2C19 , CYP2D6 , GSTT1 , GSTM1 , MTHFR , MTRR , NQO1 , NAT2 , HLA-DQA1 , and AB0 Allele Frequencies in Native Russians. *Genet Test Mol Biomarkers*; **14**: 329–342 (2010).

195 Correia C, Santos P, Coutinho AM, Vicente AM. Characterization of pharmacogenetically relevant CYP2D6 and ABCB1 gene polymorphisms in a Portuguese population sample. *Cell Biochem Funct*; **27**: 251–255 (2009).

196 Ragia G, Arvanitidis KI, Tavridou A, Manolopoulos VG. Need for reassessment of reported CYP2C19 allele frequencies in various populations in view of CYP2C19*17 discovery: the case of Greece. *Pharmacogenomics*; **10**: 43–49 (2009).

197 Bertrand‐Thiébault C *et al.* Genetic Polymorphism of CYP2C19 Gene in the Stanislas Cohort. A link with Inflammation. *Ann Hum Genet*; **72**: 178–183 (2008).

198 Crescenti A *et al.* Simultaneous genotyping of CYP2D6*3, *4, *5 AND *6 polymorphisms in a Spanish population through multiplex long polymerase chain reaction and minisequencing multiplex single base extension analysis. *Clin Exp Pharmacol Physiol*; **34**: 992–997 (2007).

199 Hilli J, Rane A, Lundgren S, Bertilsson L, Laine K. Genetic polymorphism of cytochrome P450s and P-glycoprotein in the Finnish population. *Fundam Clin Pharmacol*; **21**: 379–386 (2007).

200 Menoyo A, del Rio E, Baiget M. Characterization of variant alleles of cytochrome CYP2D6 in a Spanish population. *Cell Biochem Funct*; **24**: 381–385 (2006).

201 Rasmussen JO *et al.* CYP2D6 gene test in psychiatric patients and healthy volunteers. *Scand J Clin Lab Invest*; **66**: 129–136 (2006).

202 Halling J *et al.* Polymorphism of CYP2D6, CYP2C19, CYP2C9 and CYP2C8 in the Faroese population. *Eur J Clin Pharmacol*; **61**: 491–497 (2005).

203 Scordo MG, Caputi AP, D’Arrigo C, Fava G, Spina E. Allele and genotype frequencies of CYP2C9, CYP2C19 and CYP2D6 in an Italian population. *Pharmacol Res*; **50**: 195–200 (2004).

204 Gaikovitch EA *et al.* Polymorphisms of drug-metabolizing enzymes CYP2C9, CYP2C19, CYP2D6, CYP1A1, NAT2 and of P-glycoprotein in a Russian population. *Eur J Clin Pharmacol*; **59**: 303–312 (2003).

205 Zackrisson A-L, Lindblom B. Identification of CYP2D6 alleles by single nucleotide polymorphism analysis using pyrosequencing. *Eur J Clin Pharmacol*; **59**: 521–526 (2003).

206 Tamminga W *et al.* The prevalence of CYP2D6 and CYP2C19 genotypes in a population of healthy Dutch volunteers. *Eur J Clin Pharmacol*; **57**: 717–722 (2001).

207 Duzhak T *et al.* Genetic polymorphisms of CYP2D6, CYP1A1, GSTM1 and p53 genes in a unique Siberian population of Tundra Nentsi. *Pharmacogenetics*; **10**: 531–537 (2000).

208 Hoskins JM, Shenfield GM, Gross AS. Relationship between proguanil metabolic ratio and CYP2C19 genotype in a Caucasian population. *Br J Clin Pharmacol*; **46**: 499–504 (1998).

209 Ruas JL, Lechner MC. Allele frequency of CYP2C19 in a Portuguese population. *Pharmacogenetics*; **7**: 333–335 (1997).

210 Sachse C, Brockmöller J, Bauer S, Roots I. Cytochrome P450 2D6 variants in a Caucasian population: allele frequencies and phenotypic consequences. *Am J Hum Genet*; **60**: 284–95 (1997).

211 Akhmedova SN *et al.* CYP2D6 Genotyping in a Russian Population Using a Novel Approach for Identification of the CYP2D6A Mutation. *Biochem Mol Med*; **58**: 234–236 (1996).

212 Albuquerque J, Ribeiro C, Naranjo MEG, Llerena A, Grazina M. Characterization of CYP2D6 genotypes and metabolic profiles in the Portuguese population: pharmacogenetic implications. *Per Med*; **10**: 709–718 (2013).

213 Caccamo D *et al.* Xenobiotic sensor- and metabolism-related gene variants in environmental sensitivity-related illnesses: a survey on the Italian population. *Oxid Med Cell Longev*; **2013**: 831969 (2013).

214 Barañska M, Dziankowska-bartkowiak B, Waszczykowska E, Rychlik-sych M, Skrêtkowicz J. Significance of genetic polymorphism of CYP2D6 in the pathogenesis of systemic sclerosis. *Pharmacoglogical Reports*; **64**: 336–342 (2012).

215 Nyírő G *et al.* The effect of the CYP 2C19*2 polymorphism on stroke care. *Acta Physiol Hung*; **99**: 33–9 (2012).

216 Pedersen RS, Christensen MMH, Brøsen K. Linkage disequilibrium between the CYP2C19*17 allele and other clinically important CYP2C allelic variants in a healthy Scandinavian population. *Eur J Clin Pharmacol*; **68**: 1463–1464 (2012).

217 Rideg O *et al.* Pilot study for the characterization of pharmacogenetically relevant CYP2D6, CYP2C19 and ABCB1 gene polymorphisms in the Hungarian population. *Cell Biochem Funct*; **29**: 562–568 (2011).

218 Adrian A *et al.* Detection of CYP2D6*6 allele by real time polymerase chain reaction in Romanian population. *Farmacia*; **58**: 353–361 (2010).

219 Berg ND *et al.* Genetic susceptibility factors for multiple chemical sensitivity revisited. *Int J Hyg Environ Health*; **213**: 131–139 (2010).

220 De Luca C *et al.* Biological definition of multiple chemical sensitivity from redox state and cytokine profiling and not from polymorphisms of xenobiotic-metabolizing enzymes. *Toxicol Appl Pharmacol*; **248**: 285–292 (2010).

221 Pedersen RS *et al.* Linkage disequilibrium between the CYP2C19*17 allele and wildtype CYP2C8 and CYP2C9 alleles: Identification of CYP2C haplotypes in healthy Nordic populations. *Eur J Clin Pharmacol*; **66**: 1199–1205 (2010).

222 Buzková H, Pechandová K, Slanař O, Perlík F. Frequency of single nucleotide polymorphisms of CYP2D6 in the Czech population. *Cell Biochem Funct*; **26**: 76–81 (2008).

223 Fernández-Santander A *et al.* Identification of CYP2D6 null variants among long-stay, chronic psychiatric inpatients: Is it strictly necessary? *Hum Psychopharmacol*; **23**: 533–536 (2008).

224 Makeeva O, Stepanov V, Puzyrev V, Goldstein DB, Grossman I. Global pharmacogenetics: genetic substructure of Eurasian populations and its effect on variants of drug-metabolizing enzymes. *Pharmacogenomics*; **9**: 847–868 (2008).

225 Wijnen PAHM *et al.* Role of Cytochrome P450 Polymorphisms in the Development of Pulmonary Drug Toxicity. *Drug Saf*; **31**: 1125–1134 (2008).

226 Oliveira E *et al.* Pharmacogenetically relevant polymorphisms in Portugal. *Pharmacogenomics*; **8**: 703–12 (2007).

227 Alonso-Navarro H *et al.* CYP2C19 polymorphism and risk for essential tremor. *Eur Neurol*; **56**: 119–23 (2006).

228 Scordo M, Dahl M, Spina E, Cordici F, Arena M. No association between CYP2D6 polymorphism and Alzheimer’s disease in an Italian population. *Pharmacol Res*; **53**: 162–165 (2006).

229 Niewiński P *et al.* CYP2D6 phenotype versus genotype and the potential risk of allergic diseases. *Adv Clin Exp Med*; **14**: 1175–1180 (2005).

230 Chou W-H. Comparison of Two CYP2D6 Genotyping Methods and Assessment of Genotype-Phenotype Relationships. *Clin Chem*; **49**: 542–551 (2003).

231 Agúndez JAG *et al.* Functionally Active Duplications of the *CYP2D6* Gene Are More Prevalent among Larynx and Lung Cancer Patients. *Oncology*; **61**: 59–63 (2001).

232 Brown MA *et al.* Polymorphisms of the CYP2D6 gene increase susceptibility to ankylosing spondylitis. *Hum Mol Genet*; **9**: 1563–1566 (2000).

233 Gawronska-Szklarz B *et al.* CYP2D6 and GSTM1 genotypes in a Polish population. *Eur J Clin Pharmacol*; **55**: 389–92 (1999).

234 Yamada H *et al.* CYP2D6 and CYP2C19 genotypes in an elderly Swedish population. *Eur J Clin Pharmacol*; **54**: 479–81 (1998).

235 Marandi T, Dahl ML, Rägo L, Kiivet R, Sjöqvist F. Debrisoquine and S-mephenytoin hydroxylation polymorphisms in a Russian population living in Estonia. *Eur J Clin Pharmacol*; **53**: 257–60 (1997).

236 Ruas JL, Lechner MC. Allele frequency of CYP2C19 in a Portuguese population. *Pharmacogenetics*; **7**: 333–5 (1997).

237 Ladona M *et al.* CYP2D6 genotypes in Spanish women with breast cancer. *Cancer Lett*; **99**: 23–28 (1996).

238 Marandi T, Dahl ML, Kiivet RA, Rägo L, Sjöqvist F. Debrisoquin and S-mephenytoin hydroxylation phenotypes and CYP2D6 genotypes in an Estonian population. *Pharmacol Toxicol*; **78**: 303–7 (1996).

239 Agúndez JAG, Ledesma MC, Ladero JM, Benítez J. Prevalence of CYP2D6 gene duplication and its repercussion on the oxidative phenotype in a white population*. *Clin Pharmacol Ther*; **57**: 265–269 (1995).

240 Brockmöller J, Rost KL, Gross D, Schenkel A, Roots I. Phenotyping of CYP2C19 with enantiospecific HPLC-quantification of R- and S-mephenytoin and comparison with the intron4/exon5 G-->A-splice site mutation. *Pharmacogenetics*; **5**: 80–8 (1995).

241 Agúndez JAG *et al.* Genetic basis for differences in debrisoquin polymorphism between a spanish and other white populations. *Clin Pharmacol Ther*; **55**: 412–417 (1994).

242 Tefre T *et al.* Genotyping of the CYP2D6 gene in Norwegian lung cancer patients and controls. *Pharmacogenetics*; **4**: 47–57 (1994).

243 Graf T *et al.* Prediction of phenotype for acetylation and for debrisoquine hydroxylation by DNA-tests in healthy human volunteers. *Eur J Clin Pharmacol*; **43**: 399–403 (1992).

244 Broly F *et al.* Debrisoquine/Sparteine Hydroxylation Genotype and Phenotype: Analysis of Common Mutations and Alleles of CYP2D6 in a European Population. *DNA Cell Biol*; **10**: 545–558 (1991).

245 Mirzaev KB *et al.* Genetic Polymorphisms of Cytochrome P450 Enzymes and Transport Proteins in a Russian Population and Three Ethnic Groups of Dagestan. *Genet Test Mol Biomarkers*; **21**: 747–753 (2017).

246 Sychev D *et al.* Comparison of CYP2C9, CYP2C19, CYP2D6, ABCB1, and SLCO1B1 gene-polymorphism frequency in Russian and Nanai populations. *Pharmgenomics Pers Med*; **Volume10**: 93–99 (2017).

247 Barańska M *et al.* Genetic polymorphism of CYP2D6 in patients with systemic lupus erythematosus and systemic sclerosis. *Autoimmunity*; **6934**: 1–6 (2016).

248 Naranjo MEG *et al.* High frequency of CYP2D6 ultrarapid metabolizers in Spain: controversy about their misclassification in worldwide population studies. *Pharmacogenomics J*; **16**: 485–490 (2016).

249 Pietarinen P, Tornio A, Niemi M. High Frequency of CYP2D6 Ultrarapid Metabolizer Genotype in the Finnish Population. *Basic Clin Pharmacol Toxicol*; **119**: 291–296 (2016).

250 Rychlik-Sych M *et al.* The impact of the CYP2D6 gene polymorphism on the risk of pemphigoid. *Int J Dermatol*; **54**: 1396–1401 (2015).

251 Weber A *et al.* Increased prevalence of functional minor allele variants of drug metabolizing CYP2B6 and CYP2D6 genes in Roma population samples. *Pharmacol Reports*; **67**: 460–464 (2015).

252 Dudarewicz M *et al.* Significance of the genetic polymorphism of CYP2D6 and NAT2 in patients with inflammatory bowel diseases. *Pharmacol Reports*; **66**: 686–690 (2014).

253 Kuhlmann JB, Wensing G, Kuhlmann J. Correlation of genotype, phenotype, and mRNA expression of CYP2D6 and CYP2C19 in peripheral blood leukocytes (PBLs). *Int J Clin Pharmacol Ther*; **52**: 143–150 (2014).

254 Tervasmäki A, Winqvist R, Jukkola-Vuorinen A, Pylkäs K. Recurrent CYP2C19 deletion allele is associated with triple-negative breast cancer. *BMC Cancer*; **14**: 1–7 (2014).

255 Dahl M-L, Johansson I, Palmertz MP, Ingelman-Sundberg M, Sjöqvist F. Analysis of the CYP2D6 gene in relation to debrisoquin and desipramine hydroxylation in a Swedish population. *Clin Pharmacol Ther*; **51**: 12–17 (1992).

256 Dlugauskas E *et al.* Analysis of Lithuanian CYP2D6 polymorphism and its relevance to psychiatric care of the local population. *Nord J Psychiatry*; **73**: 31–35 (2019).

257 Sałagacka-Kubiak A, Żebrowska-Nawrocka M, Jeleń A, Mirowski M, Balcerczak E. CYP2C19*2 polymorphism in Polish peptic ulcer patients. *Pharmacol Reports*; **71**: 272–275 (2019).

258 Belmonte C *et al.* Influence of CYP2D6 , CYP3A4 , CYP3A5 and ABCB1 Polymorphisms on Pharmacokinetics and Safety of Aripiprazole in Healthy Volunteers. *Basic Clin Pharmacol Toxicol*; **122**: 596–605 (2018).

259 Reisberg S *et al.* Translating genotype data of 44,000 biobank participants into clinical pharmacogenetic recommendations: challenges and solutions. *Genet Med*; **21**: 1345–1354 (2019).

260 Zhou Y, Lauschke VM. Comprehensive overview of the pharmacogenetic diversity in Ashkenazi Jews. *J Med Genet*; **55**: 617–627 (2018).

261 Hočevar K, Maver A, Peterlin B. Actionable Pharmacogenetic Variation in the Slovenian Genomic Database. *Front Pharmacol*; **10**: 1–11 (2019).

262 Tabari RG, Marjani A, Ataby OA, Mansourian AR, Samai NM. Genetic Polymorphism of Cytochrome p450 (2C19) Enzyme in Iranian Turkman Ethnic Group. *Oman Med J*; **28**: 237–44 (2013).

263 Saeed LH, Mayet AY. Genotype-Phenotype Analysis of CYP2C19 in Healthy Saudi Individuals and its Potential Clinical Implication in Drug Therapy. *Int J Med Sci*; **10**: 1497–1502 (2013).

264 Yousef A-M *et al.* Allele and genotype frequencies of the polymorphic cytochrome P450 genes (CYP1A1, CYP3A4, CYP3A5, CYP2C9 and CYP2C19) in the Jordanian population. *Mol Biol Rep*; **39**: 9423–9433 (2012).

265 Zihlif M, Imraish A, Irshaid YM. Frequency of Certain Single-Nucleotide Polymorphisms and Duplication of CYP2D6 in the Jordanian Population. *Genet Test Mol Biomarkers*; **16**: 1201–1205 (2012).

266 Hashemi-Soteh SMB, Sarzare F, Merat F, Salehifar E, Shiran M-R. Frequencies of Three CYP2D6 Nonfunctional Alleles ( CYP2D6*3 , *4 , and *6 ) Within an Iranian Population (Mazandaran). *Genet Test Mol Biomarkers*; **15**: 821–825 (2011).

267 Djaffar Jureidini I *et al.* Prevalence of CYP2C19 polymorphisms in the Lebanese population. *Mol Biol Rep*; **38**: 5449–5452 (2011).

268 Kouhi H, Hamzeiy H, Barar J, Asadi M, Omidi Y. Frequency of Five Important CYP2D6 Alleles Within an Iranian Population (Eastern Azerbaijan). *Genet Test Mol Biomarkers*; **13**: 665–670 (2009).

269 Sameer A-EI, Amany GM, Abdela AA, Fadel SA. CYP2C19 genotypes in a population of healthy volunteers and in children with hematological malignancies in Gaza Strip. *Can J Clin Pharmacol*; **16**: e156-62 (2009).

270 Koseler A, Ilcol YO, Ulus IH. Frequency of Mutated Allele CYP2D6*4 in the Turkish Population. *Pharmacology*; **79**: 203–206 (2007).

271 Zand N, Tajik N, Moghaddam AS, Milanian I. Genetic polymorphisms of cytochrome P450 enzymes 2C9 and 2C19 in a healthy Iranian population. *Clin Exp Pharmacol Physiol*; **34**: 102–105 (2007).

272 Aydin M, Hatirnaz O, Erensoy N, Ozbek U. CYP2D6 and CYP1A1 mutations in the Turkish population. *Cell Biochem Funct*; **23**: 133–135 (2005).

273 Luo H, Aloumanis V, Lin K, Gurwitz D, Wan YY. Polymorphisms of CYP2C19 and CYP2D6 in Israeli ethnic groups. *Am J Pharmacogenomics*; **4**: 395–401 (2004).

274 Aynacioglu A *et al.* Low frequency of defective alleles of cytochrome P450 enzymes 2C19 and 2D6 in the Turkish population. *Clin Pharmacol Ther*; **66**: 185–192 (1999).

275 Sviri S, Shpizen S, Leitersdorf E, Levy M, Caraco Y. Phenotypic-genotypic analysis of CYP2C19 in the Jewish Israeli population. *Clin Pharmacol Ther*; **65**: 275–282 (1999).

276 Nassar S *et al.* ABCB1 C3435T and CYP2C19*2 polymorphisms in a Palestinian and Turkish population: A pharmacogenetic perspective to clopidogrel. *Meta Gene*; **2**: 314–319 (2014).

277 Al-Dosari MS *et al.* High prevalence of CYP2D6*41 (G2988A) allele in Saudi Arabians. *Environ Toxicol Pharmacol*; **36**: 1063–1067 (2013).

278 Al-Jenoobi FI *et al.* CYP2C19 Genetic Polymorphism in Saudi Arabians. *Basic Clin Pharmacol Toxicol*; **112**: 50–54 (2013).

279 Serin A, Canan H, Alper B, Gulmen. M. The frequencies of mutated alleles of CYP2D6 gene in a Turkish population. *Forensic Sci Int*; **222**: 332–334 (2012).

280 Erden G *et al.* Frequency of mutated allele CYP2D6*4 in the Turkish ankylosing spondylitis patients and healthy controls. *Rheumatol Int*; **29**: 1431–1434 (2009).

281 Gutman G *et al.* CYP1A1 and CYP2D6 Gene Polymorphisms in Israeli Jewish Women With Cervical Cancer. *Int J Gynecol Cancer*; **19**: 1300–1302 (2009).

282 Homaei-Shandiz F *et al.* Study the frequency of CYP2D6*4 null allele in Iranian population. *Pharmacol 3*; **3**: 1003–1007 (2009).

283 Kortunay S *et al.* CYP2C19 genotype does not represent a genetic predisposition in idiopathic systemic lupus erythematosus. *Ann Rheum Dis*; **58**: 182–185 (1999).

284 McLellan RA, Oscarson M, Seidegård J, Evans DA, Ingelman-Sundberg M. Frequent occurrence of CYP2D6 gene duplication in Saudi Arabians. *Pharmacogenetics*; **7**: 187–91 (1997).

285 Hadidi HF *et al.* Debrisoquine 4-hydroxylation (CYP2D6) polymorphism in Jordanians. *Pharmacogenetics*; **4**: 159–61 (1994).

286 Arici M, Özhan G. CYP2C9, CYPC19 and CYP2D6 gene profiles and gene susceptibility to drug response and toxicity in Turkish population. *Saudi Pharm J*; **25**: 376–380 (2017).

287 Barlas İÖ *et al.* Harnessing Knowledge on Very Important Pharmacogenes CYP2C9 and CYP2C19 Variation for Precision Medicine in Resource-Limited Global Conflict Zones. *Omi A J Integr Biol*; **20**: 604–609 (2016).

288 Ghasemi Z *et al.* Development of a high-resolution melting analysis method for CYP2C19*17 genotyping in healthy volunteers. *Avicenna J Med Biotechnol*; **8**: 193–199 (2016).

289 Taskin B, Percin FE, Ergun MA. Investigation of CYP2D6 Gene Polymorphisms in Turkish Population. *Psychopharmacol Bull*; **46**: 67–72 (2016).

290 Yasin S *et al.* Screening for CYP2C19 Gene variants in a healthy Jordanian population. *Trop J Pharm Res*; **15**: 2745 (2017).

291 Kamalidehghan B *et al.* Prevalence of the CYP2D6*10(C100T), *4(G1846A), and *14(G1758A) alleles among Iranians of different ethnicities. *Drug Des Devel Ther*; : 2627 (2015).

292 Payan M, Tajik N, Rouini MR, Ghahremani MH. Genotype and allele frequency of CYP2C19*17 in a healthy Iranian population. *Med J Islam Repub Iran*; **29**: 269 (2015).

293 Uckun Z *et al.* The impact of CYP2C19 polymorphisms on citalopram metabolism in patients with major depressive disorder. *J Clin Pharm Ther*; **40**: 672–679 (2015).

294 Dehbozorgi M *et al.* Prevalence of the CYP2C19*2 (681 G&gt;A), *3 (636 G&gt;A) and *17 (‑806 C&gt;T) alleles among an Iranian population of different ethnicities. *Mol Med Rep*; **17**: 4195–4202 (2018).

295 Hashemizadeh Z, Malek-Hosseini SA, Badiee P. Prevalence of CYP2C19 Genetic Polymorphism among Normal People and Patients with Hepatic Diseases. *Int J organ Transplant Med*; **9**: 27–33 (2018).

296 Lotfi F *et al.* Cytochrome P450 (CYP450,2D6*A), N-Acetyltransferase-2 (NAT2*7, A) and Multidrug Resistance 1 (MDR1 3435 T) Alleles Collectively Increase Risk of Ulcerative Colitis. *Arch Iran Med*; **21**: 530–535 (2018).

297 Pourkarami Z *et al.* Genetic polymorphisms of CYP2D6*41 in different ethnicities of Iranian population. *Gazz Med Ital*; **177**: 158–164 (2018).

298 Hsu H-L, Woad KJ, Woodfield DG, Helsby NA. A high incidence of polymorphic CYP2C19 variants in archival blood samples from Papua New Guinea. *Hum Genomics*; **3**: 17 (2008).

299 Griese E-U *et al.* Allele and genotype frequencies of polymorphic cytochromes P4502D6, 2C19 and 2E1 in Aborigines from Western Australia. *Pharmacogenetics*; **11**: 69–76 (2001).

300 Kaneko A *et al.* High and variable frequencies of CYP2C19 mutations: medical consequences of poor drug metabolism in Vanuatu and other Pacific islands. *Pharmacogenetics*; **9**: 581–90 (1999).
