# Supplementary material for: Meta-analysis of probability estimates of worldwide variation of CYP2D6 and CYP2C19
Source: Transl Psychiatry. 2021 Feb 24;11:141. doi: 10.1038/s41398-020-01129-1 (PMC7904867; doi:10.1038/s41398-020-01129-1)
Supplement: Supplementary file 3 — Supplemental Table 2: References for Table 3 [file 41398_2020_1129_MOESM3_ESM.doc]

| **Supplemental Table 2: References for Table 3** | |
| --- | --- |
| ***Africa*** |  |
| African | 1 |
| African (north and East) | 2 |
| Biaka, Pygmies (Subsaharan Africa) | 3 |
| Colored (South African) | 4,5 |
| Esan (Nigerian) | 6 |
| Gambian | 6 |
| Luhya in Webuye (Kenian) | 6 |
| Mandenka (Subsaharan Africa) | 3 |
| Mende (Sierra Leonean) | 6 |
| Mozabite (Algerian) | 3 |
| North African | 7 |
| South African | 8 |
| West African | 7 |
| Xhosa (South African) | 9 |
| Yoruba (Nigerian) (Ibadan) | 6 |
| Yoruba (Subsaharan Africa) | 3 |
| ***African American*** | 6,10,11 |
|  |  |
| ***Americas*** |  |
| Admixed Latin Americans | 1,12 |
| African Caribbean (Barbados) | 6 |
| African Caribbean from Barbados (Costa Rica) | 13 |
| Afro-Latin Americans | 12 |
| American Indian | 14 |
| Brazilian (North East) | 15 |
| Brazilian (North) | 15 |
| Brazilian (South East) | 15 |
| Brazilian (South) | 15 |
| Brazilian from African descent | 16 |
| Brazilian from European descent | 16 |
| Central Native Americans | 12 |
| Colombian | 6,17 |
| Coras (Mexican) | 18 |
| Cuban from European descent | 19 |
| Dutch Caribbean | 20 |
| Ecuadorian | 21,22 |
| Huicholes (Mexican) | 18 |
| Iberians | 12 |
| Indo-Trinidad | 13 |
| Jewish (Ashkenazi) | 12 |
| Karitiana (South American) | 3 |
| Lacandones (Mexican) | 23 |
| Maya (Mexican) | 18 |
| Maya (South American) | 3 |
| Mestizo (Cuban) | 19 |
| Mestizo (Mexican) | 23–25 |
| Mestizo (Nicarguan) | 19,26 |
| Mexican | 6,18,27,28 |
| North Native Americans | 12 |
| Peruvian | 6 |
| Pima (South American) | 3 |
| Puerto Rican | 6 |
| South American | 7 |
| South Native Americans | 12 |
| Surui (South American) | 3 |
| Tarahumares (Mexican) | 18 |
| Tepehuanos (Mexican) | 18 |
| White Latin Americans | 12 |
|  |  |
| ***Central/ South East Asia*** |  |
| Balochi (Central/South Asia) | 3 |
| Bengali | 6 |
| Brahui (Central/South Asia) | 3 |
| Burusho (Central/South Asia) | 3 |
| Gujarati (Indian) | 6 |
| Hazara (Central/South Asia) | 3 |
| Kalash (Central/South Asia) | 3 |
| Makrani (Central/South Asia) | 3 |
| Pathan (Central/South Asia) | 3 |
| Punjabi (Indian) | 6 |
| Sindhi (Central/South Asia) | 3 |
| South Asian | 1,7 |
| South East Asia | 7 |
| Tamil (Sri Lankan) | 6 |
| Telugu (Indian) | 6 |
| Uyghurs (Central/South Asia) | 3 |
| Viet Kinh (Vietnamese) | 6 |
| Western India | 29 |
|  |  |
| ***East Asia*** |  |
| Chinese | 30–32 |
| Dai (Chinese) | 6 |
| East Asian | 1 |
| Han (Chinese) | 6,33–35 |
| Han (East Asia) | 3 |
| Japanese | 3,6,30,36,37 |
| Shanghai (Chinese) | 31 |
| Shantou (Chinese) | 31 |
| Shenyang (Chinese) | 31 |
| South Korean | 30,38–40 |
| Tibetan Chinese | 41 |
| Xi’an (Chinese) | 31 |
| Yakut (East Asia) | 3 |
|  |  |
| ***Europe*** |  |
| Basque (French) | 3 |
| British | 6 |
| European (Danish) | 42 |
| European | 1,43 |
| European (Hungarian) | 44 |
| European (USA) | 6,45 |
| Estonian | 46 |
| Finnish | 6 |
| Finnish (East) | 29 |
| Finnish (West) | 29 |
| French | 3 |
| German | 47 |
| Iberian (Spanish) | 6 |
| Jewish (Ashkenazi) | 43 |
| Lithuania | 48 |
| Ravenna Italian | 2 |
| Roma (Italian) | 7 |
| Russian | 3 |
| Sardinian (Italian) | 3 |
| Spanish | 49 |
| Toscani (Italian) | 6 |
|  |  |
| ***Middle East*** |  |
| Arabian Bedouins (Israel) | 50 |
| Bedouin (Middle Eastern) | 3 |
| Druze (Middle Eastern) | 3 |
| Jewish (Israel) | 50 |
| Palestinian (Westbank) | 3 |
|  |  |
| ***Oceania*** |  |
| Aboriginal (North West Australia) | 51 |
| non-Austronesian Melanesian | 3 |

1 Zhou Y *et al.* Worldwide Distribution of Cytochrome P450 Alleles: A Meta-analysis of Population-scale Sequencing Projects. *Genet Med*; **21**: 1345–1354 (2019).

2 Riccardi LN *et al.* CYP2D6 polymorphism studies: How forensic genetics helps clinical medicine. *Forensic Sci Int Genet Suppl Ser*; **2**: 485–486 (2009).

3 Sistonen J *et al.* CYP2D6 worldwide genetic variation shows high frequency of altered activity variants and no continental structure. *Pharmacogenet Genomics*; **17**: 93–101 (2007).

4 Gaedigk A, Coetsee C. The CYP2D6 gene locus in South African Coloureds: unique allele distributions, novel alleles and gene arrangements. *Eur J Clin Pharmacol*; **64**: 465–475 (2008).

5 Dodgen TM *et al.* Pharmacogenetic comparison of CYP2D6 predictive and measured phenotypes in a South African cohort. *Pharmacogenomics J*; **16**: 566–572 (2016).

6 Wendt FR, Pathak G, Sajantila A, Chakraborty R, Budowle B. Global genetic variation of select opiate metabolism genes in self-reported healthy individuals. *Pharmacogenomics J*; **18**: 281–294 (2018).

7 Riccardi LN *et al.* CYP2D6 Genotyping in Natives and Immigrants from the Emilia-Romagna Region (Italy). *Genet Test Mol Biomarkers*; **15**: 801–806 (2011).

8 Dodgen TM *et al.* Introduction of the AmpliChip CYP450 Test to a South African cohort: a platform comparative prospective cohort study. *BMC Med Genet*; **14**: 20 (2013).

9 Wright GEB *et al.* Elucidation of CYP2D6 Genetic Diversity in a Unique African Population: Implications for the Future Application of Pharmacogenetics in the Xhosa Population. *Ann Hum Genet*; **74**: 340–350 (2010).

10 Gaedigk A, Bradford LD, Marcucci K a, Leeder JS. Unique CYP2D6 activity distribution and genotype-phenotype discordance in black Americans. *Clin Pharmacol Ther*; **72**: 76–89 (2002).

11 Cai W-M *et al.* CYP2D6 genetic variation in healthy adults and psychiatric African-American subjects: implications for clinical practice and genetic testing. *Pharmacogenomics J*; **6**: 343–350 (2006).

12 Naranjo M-EG *et al.* Interethnic Variability in CYP2D6, CYP2C9, and CYP2C19 Genes and Predicted Drug Metabolism Phenotypes Among 6060 Ibero- and Native Americans: RIBEF-CEIBA Consortium Report on Population Pharmacogenomics. *Omi A J Integr Biol*; **22**: 575–588 (2018).

13 Montané Jaime LK, Lalla A, Steimer W, Gaedigk A. Characterization of the CYP2D6 gene locus and metabolic activity in Indo- and Afro-Trinidadians: discovery of novel allelic variants. *Pharmacogenomics*; **14**: 261–76 (2013).

14 Fohner A *et al.* Pharmacogenetics in American Indian populations. *Pharmacogenet Genomics*; **23**: 403–414 (2013).

15 Friedrich DC *et al.* Distribution of CYP2D6 Alleles and Phenotypes in the Brazilian Population. *PLoS One*; **9**: e110691 (2014).

16 Kohlrausch FB *et al.* Molecular diversity at the CYP2D6 locus in healthy and schizophrenic southern Brazilians. *Pharmacogenomics*; **10**: 1457–1466 (2009).

17 Isaza CA, Henao J, López AM, Cacabelos R. Isolation, sequence and genotyping of the drug metabolizer CYP2D6 gene in the Colombian population. *Methods Find Exp Clin Pharmacol*; **22**: 695–705 (2000).

18 Lazalde-Ramos BP *et al.* CYP2D6 gene polymorphisms and predicted phenotypes in eight indigenous groups from northwestern Mexico. *Pharmacogenomics*; **15**: 339–348 (2014).

19 LLerena A *et al.* CYP2D6 genotype and debrisoquine hydroxylation phenotype in Cubans and Nicaraguans. *Pharmacogenomics J*; **12**: 176–183 (2012).

20 Koopmans AB, Vinkers DJ, Gelan PJ, Hoek HW, van Harten PN. CYP2D6 and CYP2C19 genotyping in psychiatric patients on psychotropic medication in the former Dutch Antilles. *Pharmacogenomics*; **18**: 1003–1012 (2017).

21 Dorado P *et al.* CYP2D6 genotype and dextromethorphan hydroxylation phenotype in an Ecuadorian population. *Eur J Clin Pharmacol*; **68**: 637–644 (2012).

22 de Andrés F, Terán S, Hernández F, Terán E, LLerena A. To genotype or phenotype for personalized medicine? CYP450 drug metabolizing enzyme genotype–phenotype concordance and discordance in the Ecuadorian population. *Omi A J Integr Biol*; **20**: 699–710 (2016).

23 López-López M *et al.* CYP2D6 genetic polymorphisms in Southern Mexican Mayan Lacandones and Mestizos from Chiapas. *Pharmacogenomics*; **15**: 1859–1865 (2014).

24 Contreras A V *et al.* Resequencing, haplotype construction and identification of novel variants of CYP2D6 in Mexican Mestizos. *Pharmacogenomics*; **12**: 745–756 (2011).

25 López M, Guerrero J, Jung–Cook H, Alonso ME. CYP2D6 genotype and phenotype determination in a Mexican Mestizo population. *Eur J Clin Pharmacol*; **61**: 749–754 (2005).

26 Agúndez JA, Ramirez R, Hernandez M, Llerena A, Benítez J. Molecular heterogeneity at the CYP2D gene locus in Nicaraguans: impact of gene-flow from Europe. *Pharmacogenetics*; **7**: 337–40 (1997).

27 Luo H-R, Gaedigk A, Aloumanis V, Wan Y-JY. Identification of CYP2D6 impaired functional alleles in Mexican Americans. *Eur J Clin Pharmacol*; **61**: 797–802 (2005).

28 de Andrés F, Sosa-Macías M, Ramos BPL, Naranjo M-EG, LLerena A. CYP450 Genotype/Phenotype Concordance in Mexican Amerindian Indigenous Populations–Where to from Here for Global Precision Medicine? *Omi A J Integr Biol*; **21**: 509–519 (2017).

29 Sistonen J *et al.* Pharmacogenetic variation at CYP2C9, CYP2C19, and CYP2D6 at global and microgeographic scales. *Pharmacogenet Genomics*; **19**: 170–179 (2009).

30 Myrand S *et al.* Pharmacokinetics/Genotype Associations for Major Cytochrome P450 Enzymes in Native and First- and Third-generation Japanese Populations: Comparison With Korean, Chinese, and Caucasian Populations. *Clin Pharmacol Ther*; **84**: 347–361 (2008).

31 Qin S *et al.* Systematic polymorphism analysis of the CYP2D6 gene in four different geographical Han populations in mainland China. *Genomics*; **92**: 152–158 (2008).

32 Ismail R, Teh LK, Amir J, Alwi Z, Lopez CG. Genetic polymorphism of CYP2D6 in Chinese subjects in Malaysia. *J Clin Pharm Ther*; **28**: 279–84 (2003).

33 Kim E *et al.* Robust CYP2D6 genotype assay including copy number variation using multiplex single-base extension for Asian populations. *Clin Chim Acta*; **411**: 2043–2048 (2010).

34 Zhou Q *et al.* Genetic polymorphism, linkage disequilibrium, haplotype structure and novel allele analysis of CYP2C19 and CYP2D6 in Han Chinese. *Pharmacogenomics J*; **9**: 380–394 (2009).

35 Ji L *et al.* Single-step assays to analyze CYP2D6 gene polymorphisms in Asians: allele frequencies and a novel *14B allele in mainland Chinese. *Clin Chem*; **48**: 983–8 (2002).

36 Iwashima K *et al.* No association between CYP2D6 polymorphisms and personality trait in Japanese. *Br J Clin Pharmacol*; **64**: 96–99 (2007).

37 Kato D *et al.* Effects of CYP2D6 polymorphisms on neuroleptic malignant syndrome. *Eur J Clin Pharmacol*; **63**: 991–996 (2007).

38 Lee S-J *et al.* Discovery of Novel Functional Variants and Extensive Evaluation of CYP2D6 Genetic Polymorphisms in Koreans. *Drug Metab Dispos*; **37**: 1464–1470 (2009).

39 Lee S-Y *et al.* Sequence-based CYP2D6 Genotyping in the Korean Population. *Ther Drug Monit*; **28**: 382–387 (2006).

40 Byeon J-Y *et al.* CYP2D6 allele frequencies in Korean population, comparison with East Asian, Caucasian and African populations, and the comparison of metabolic activity of CYP2D6 genotypes. *Arch Pharm Res*; **41**: 921–930 (2018).

41 Jin T *et al.* Polymorphisms and phenotypic analysis of cytochrome P450 2D6 in the Tibetan population. *Gene*; **527**: 360–365 (2013).

42 Rasmussen JO *et al.* CYP2D6 gene test in psychiatric patients and healthy volunteers. *Scand J Clin Lab Invest*; **66**: 129–136 (2006).

43 Zhou Y, Lauschke VM. Comprehensive overview of the pharmacogenetic diversity in Ashkenazi Jews. *J Med Genet*; **55**: 617–627 (2018).

44 Rideg O *et al.* Pilot study for the characterization of pharmacogenetically relevant CYP2D6, CYP2C19 and ABCB1 gene polymorphisms in the Hungarian population. *Cell Biochem Funct*; **29**: 562–568 (2011).

45 Chou W-H. Comparison of Two CYP2D6 Genotyping Methods and Assessment of Genotype-Phenotype Relationships. *Clin Chem*; **49**: 542–551 (2003).

46 Reisberg S *et al.* Translating genotype data of 44,000 biobank participants into clinical pharmacogenetic recommendations: challenges and solutions. *Genet Med*; **21**: 1345–1354 (2019).

47 Stingl JC *et al.* Genetic variation in CYP2D6 impacts neural activation during cognitive tasks in humans. *Neuroimage*; **59**: 2818–2823 (2012).

48 Dlugauskas E *et al.* Analysis of Lithuanian CYP2D6 polymorphism and its relevance to psychiatric care of the local population. *Nord J Psychiatry*; **73**: 31–35 (2019).

49 Naranjo MEG *et al.* High frequency of CYP2D6 ultrarapid metabolizers in Spain: controversy about their misclassification in worldwide population studies. *Pharmacogenomics J*; **16**: 485–490 (2016).

50 Luo H, Aloumanis V, Lin K, Gurwitz D, Wan YY. Polymorphisms of CYP2C19 and CYP2D6 in Israeli ethnic groups. *Am J Pharmacogenomics*; **4**: 395–401 (2004).

51 Griese E-U *et al.* Allele and genotype frequencies of polymorphic cytochromes P4502D6, 2C19 and 2E1 in Aborigines from Western Australia. *Pharmacogenetics*; **11**: 69–76 (2001).
