# Supplementary material for: Meta-analysis of probability estimates of worldwide variation of CYP2D6 and CYP2C19
Source: Transl Psychiatry. 2021 Feb 24;11:141. doi: 10.1038/s41398-020-01129-1 (PMC7904867; doi:10.1038/s41398-020-01129-1)
Supplement: Supplementary file 4 — Supplemental Table 3: References for Table 4 [file 41398_2020_1129_MOESM4_ESM.doc]

| **Supplemental Table 3: References for Table 4** | |
| --- | --- |
| ***Africa*** |  |
| African | 1 |
| Cape Mixed Ancestry (South African) | 2 |
| Tzotziles (Ugandese) | 3 |
| Xhosa (South African) | 2 |
|  |  |
| ***African American*** | 5, 45 |
|  |  |
| ***Americas*** |  |
| Admixed Latin Americans | 1 |
| African Caribbeans from Barbados (Costa Rica) | 6 |
| Brazilian | 7 |
| Bribri (Costa Rican) | 6 |
| Costa Rican | 6 |
| Dutch Caribbean | 8 |
| Ecuadorian | 9 |
| Guarani (Brazil) | 10 |
| Guaymi (Costa Rican) | 6 |
| Mestizo (Costa Rican) | 6 |
| Mestizo (Ecuadorian) | 11 |
| Mestizo (Mexican) | 12 |
| Mexican | 13,14 |
|  |  |
| ***Central/ South East Asia*** |  |
| Buruhi (Pakistani) | 15 |
| Burushu (Pakistani) | 15 |
| Hazara (Pakistani) | 15 |
| Indian (West) | 16 |
| Kalash (Pakistani) | 15 |
| Koya (Indian) | 17 |
| Naik (Indian) | 17 |
| Pakistani | 18,19 |
| Parsi (Pakistani) | 15 |
| Pathan (Pakistani) | 15 |
| Punjabi (Pakistani) | 15 |
| Saraiki (Pakistani) | 15 |
| Sindhi (Pakistani) | 15 |
| South Asian | 1 |
| Tamil (Indian) (South) | 20 |
|  |  |
| ***East Asia*** |  |
| Bai (Chinese) | 21 |
| Chinese | 22–25 |
| East Asian | 1,5,26,27 |
| Hakka (Chinese) | 28 |
| Han (Chinese) | 21,29–34 |
| Han (Chinese) (North) | 35 |
| Han (Chinese) (South) | 35 |
| Han (Taiwanese) | 36 |
| Hui (Chinese) | 30,31 |
| Japanese | 26 |
| Japanese | 22,25,45,37–44 |
| Kazakh (Chinese) | 32 |
| Li (Chinese) | 46,47 |
| Mongolian | 30,31,48 |
| Shanghai (Chinese) | 49 |
| Shantou (Chinese) | 49 |
| Shenyang (Chinese) | 49 |
| South Korean | 22,26,50–55 |
| Tibetan Chinese | 56 |
| Tohoku (Japanese) | 43 |
| Uyghurs (Chinese) | 31,32,57 |
| Xi’an (Chinese) | 49 |
|  |  |
| ***Europe*** |  |
| Avars (Russian) | 58 |
| Danish | 59 |
| Dargins (Russian) | 58 |
| European | 1,22,60 |
| European (USA) | 4,5 |
| Finnish (North) | 61 |
| Greek | 62 |
| Hispanic (USA) | 4,5 |
| Jewish | 4 |
| Jewish (Ashkenazi) | 60,63 |
| Jewish (Sephardi) | 63 |
| Laks (Russian) | 58 |
| Macedonian | 64 |
| Nanai (Russian) | 65 |
| Norwegian | 59 |
| Scandinavian | 59 |
| Spanish (North) | 11 |
| Swedish | 52 |
|  |  |
| ***Middle East*** |  |
| Caspian (Iranian) | 66 |
| Fars (Iranian) | 66 |
| Iranian | 67,68 |
| Kurd (Iranian) | 66 |
| Lure (Iranian) | 66 |
| Saoudi Arabian | 69,70 |
| Turk (Iranian) | 66 |
| Turkish | 71,72 |
|  |  |
| ***Oceania*** |  |
| Pacific Islander | 5 |

1 Zhou Y *et al.* Worldwide Distribution of Cytochrome P450 Alleles: A Meta-analysis of Population-scale Sequencing Projects. *Genet Med*; **21**: 1345–1354 (2019).

2 Drögemöller BI *et al.* Characterization of the genetic profile of CYP2C19 in two South African populations. *Pharmacogenomics*; **11**: 1095–1103 (2010).

3 Miura J, Obua C, Abbo C, Kaneko S, Tateishi T. Cytochrome P450 2C19 genetic polymorphisms in Ugandans. *Eur J Clin Pharmacol*; **65**: 319–320 (2009).

4 Martis S *et al.* Multi-ethnic distribution of clinically relevant CYP2C genotypes and haplotypes. *Pharmacogenomics J*; **13**: 369–377 (2013).

5 Budd WT *et al.* Next generation sequencing reveals disparate population frequencies among cytochrome P450 genes: clinical pharmacogenomics of the CYP2 family. *Int J Comput Biol Drug Des*; **9**: 54 (2016).

6 Céspedes-Garro C *et al.* Relevance of the ancestry for the variability of the Drug-Metabolizing Enzymes CYP2C9, CYP2C19 and CYP2D6 polymorphisms in a multiethnic Costa Rican population. *Rev Biol Trop*; **64** (2016). doi:10.15517/rbt.v64i3.20901.

7 Suarez-Kurtz G *et al.* Global pharmacogenomics: Impact of population diversity on the distribution of polymorphisms in the CYP2C cluster among Brazilians. *Pharmacogenomics J*; **12**: 267–276 (2012).

8 Koopmans AB, Vinkers DJ, Gelan PJ, Hoek HW, van Harten PN. CYP2D6 and CYP2C19 genotyping in psychiatric patients on psychotropic medication in the former Dutch Antilles. *Pharmacogenomics*; **18**: 1003–1012 (2017).

9 de Andrés F, Terán S, Hernández F, Terán E, LLerena A. To genotype or phenotype for personalized medicine? CYP450 drug metabolizing enzyme genotype–phenotype concordance and discordance in the Ecuadorian population. *Omi A J Integr Biol*; **20**: 699–710 (2016).

10 Vargens DD, Petzl-Erler M-L, Suarez-Kurtz G. Distribution of CYP2C Polymorphisms in an Amerindian Population of Brazil. *Basic Clin Pharmacol Toxicol*; **110**: 396–400 (2012).

11 Vicente J *et al.* Genetic polymorphisms of CYP2C8, CYP2C9 and CYP2C19 in Ecuadorian Mestizo and Spaniard populations: a comparative study. *Mol Biol Rep*; **41**: 1267–1272 (2014).

12 Ortega-Vázquez A *et al.* CYP2C9, CYP2C19, ABCB1 genetic polymorphisms and phenytoin plasma concentrations in Mexican-Mestizo patients with epilepsy. *Pharmacogenomics J*; **16**: 286–292 (2016).

13 de Andrés F, Sosa-Macías M, Ramos BPL, Naranjo M-EG, LLerena A. CYP450 Genotype/Phenotype Concordance in Mexican Amerindian Indigenous Populations–Where to from Here for Global Precision Medicine? *Omi A J Integr Biol*; **21**: 509–519 (2017).

14 Favela‐Mendoza AF *et al.* Correspondence between the CYP2C19 and CYP3A4 genotypes with the inferred metabolizer phenotype by omeprazole administration in Mexican healthy children. *J Clin Pharm Ther*; **43**: 656–663 (2018).

15 Riaz S *et al.* Genetic Polymorphism of CYP2C19 in Pakistani Population. *Iran J Pharm Res*; **18**: 1097–1102 (2019).

16 Shalia KK, Shah VK, Pawar P, Divekar SS, Payannavar S. Polymorphisms of MDR1, CYP2C19 and P2Y12 genes in Indian population: Effects on clopidogrel response. *Indian Heart J*; **65**: 158–167 (2013).

17 Deshpande N *et al.* Rapid and ultra-rapid metabolizers with CYP2C19 *17 polymorphism do not respond to standard therapy with proton pump inhibitors. *Meta Gene*; **9**: 159–164 (2016).

18 Afsar NA *et al.* Implications of genetic variation of common Drug Metabolizing Enzymes and ABC Transporters among the Pakistani Population. *Sci Rep*; **9**: 7323 (2019).

19 Riaz S *et al.* Association of CYP2C19 * 2 and * 17 genetic variants with hypertension in Pakistani population. *Trop J Pharm Res*; **18**: 851–855 (2019).

20 Anichavezhi D, Chakradhara Rao US, Shewade DG, Krishnamoorthy R, Adithan C. Distribution of CYP2C19*17 allele and genotypes in an Indian population. *J Clin Pharm Ther*; **37**: 313–318 (2012).

21 Xiao ZS *et al.* Differences in the incidence of the CYP2C19 polymorphism affecting the S-mephenytoin phenotype in Chinese Han and Bai populations and identification of a new rare CYP2C19 mutant allele. *J Pharmacol Exp Ther*; **281**: 604–9 (1997).

22 Myrand S *et al.* Pharmacokinetics/Genotype Associations for Major Cytochrome P450 Enzymes in Native and First- and Third-generation Japanese Populations: Comparison With Korean, Chinese, and Caucasian Populations. *Clin Pharmacol Ther*; **84**: 347–361 (2008).

23 Yang YS *et al.* Genetic polymorphism of cytochrome P450 2C19 in healthy Malaysian subjects. *Br J Clin Pharmacol*; **58**: 332–335 (2004).

24 Nowak MP, Sellers EM, Tyndale RF. Canadian Native Indians exhibit unique CYP2A6 and CYP2C19 mutant allele frequencies*. *Clin Pharmacol Ther*; **64**: 378–383 (1998).

25 Goldstein JA *et al.* Frequencies of the defective CYP2C19 alleles responsible for the mephenytoin poor metabolizer phenotype in various Oriental, Caucasian, Saudi Arabian and American black populations. *Pharmacogenetics*; **7**: 59–64 (1997).

26 Man M *et al.* Genetic Variation in Metabolizing Enzyme and Transporter Genes: Comprehensive Assessment in 3 Major East Asian Subpopulations With Comparison to Caucasians and Africans. *J Clin Pharmacol*; **50**: 929–940 (2010).

27 Luo H, Poland R, Lin K, Wan Y. Genetic polymorphism of cytochrome P450 2C19 in Mexican Americans: A cross-ethnic comparative study. *Clin Pharmacol Ther*; **80**: 33–40 (2006).

28 Zhong Z *et al.* Analysis of CYP2C19 Genetic Polymorphism in a Large Ethnic Hakka Population in Southern China. *Med Sci Monit*; **23**: 6186–6192 (2017).

29 Xiuchun Y, Fan L, Jingchao L, Bing X, Wei C. GW24-e1197 Allele and genotype frequencies of CYP2C19 in Chinese Han population. *Heart*; **99**: e128 (2013).

30 Yin S-J *et al.* Differences in genotype and allele frequency distributions of polymorphic drug metabolizing enzymes CYP2C19 and CYP2D6 in mainland Chinese Mongolian, Hui and Han populations. *J Clin Pharm Ther*; **37**: 364–369 (2012).

31 Zuo J, Xia D, Jia L, Guo T. Genetic polymorphisms of drug-metabolizing phase I enzymes CYP3A4, CYP2C9, CYP2C19 and CYP2D6 in Han, Uighur, Hui and Mongolian Chinese populations. *Pharmazie*; **67**: 639–644 (2011).

32 Wang S-M *et al.* Frequencies of genotypes and alleles of the functional SNPs in CYP2C19 and CYP2E1 in mainland Chinese Kazakh, Uygur and Han populations. *J Hum Genet*; **54**: 372–375 (2009).

33 Zhou Q *et al.* Genetic polymorphism, linkage disequilibrium, haplotype structure and novel allele analysis of CYP2C19 and CYP2D6 in Han Chinese. *Pharmacogenomics J*; **9**: 380–394 (2009).

34 Dong Y *et al.* Analysis of genetic variations in CYP2C9, CYP2C19, CYP2D6 and CYP3A5 genes using oligonucleotide microarray. *Int J Clin Exp Med*; **8**: 18917–26 (2015).

35 Hu L-M *et al.* Genetic polymorphisms and novel allelic variants of CYP2C19 in the Chinese Han population. *Pharmacogenomics*; **13**: 1571–1581 (2012).

36 Liou Y-H, Lin C-T, Wu Y-J, Wu LS-H. The high prevalence of the poor and ultrarapid metabolite alleles of CYP2D6, CYP2C9, CYP2C19, CYP3A4, and CYP3A5 in Taiwanese population. *J Hum Genet*; **51**: 857–863 (2006).

37 Perini JA *et al.* Pharmacogenetic polymorphisms in Brazilian-born, first-generation Japanese descendants. *Brazilian J Med Biol Res*; **42**: 1179–84 (2009).

38 Sugimoto K, Uno T, Yamazaki H, Tateishi T. Limited frequency of the CYP2C19*17 allele and its minor role in a Japanese population. *Br J Clin Pharmacol*; **65**: 437–439 (2008).

39 Ishii G, Suzuki A, Oshino S, Shiraishi H, Otani K. CYP2C19 polymorphism affects personality traits of Japanese females. *Neurosci Lett*; **411**: 77–80 (2007).

40 Fukushima-Uesaka H *et al.* Genetic variations and haplotypes of CYP2C19 in a Japanese population. *Drug Metab Pharmacokinet*; **20**: 300–7 (2005).

41 Kimura M, Ieiri I, Mamiya K, Urae A, Higuchi S. Genetic polymorphism of cytochrome P450s, CYP2C19, and CYP2C9 in a Japanese population. *Ther Drug Monit*; **20**: 243–7 (1998).

42 Kubota T, Chiba K, Ishizaki T. Genotyping of S-mephenytoin 4’-hydroxylation in an extended Japanese population. *Clin Pharmacol Ther*; **60**: 661–6 (1996).

43 Ohkubo T, Suno M, Sugawara K, Motomuro S. Graphic Roots of CYP2C19 genetic polymorphism in Japanese population (preliminary report). *Int Congr Ser*; **1244**: 63–67 (2002).

44 Takakubo F, Kuwano A, Kondo I. Evidence that poor metabolizers of (S)-mephenytoin could be identified by haplotypes of CYP2C19 in Japanese. *Pharmacogenetics*; **6**: 265–267 (1996).

45 Tsuneoka Y, Fukushima K, Matsuo Y, Ichikawa Y, Watanabe Y. Genotype analysis of the CYP2C19 gene in the Japanese population. *Life Sci*; **59**: 1711–5 (1996).

46 Wang J-H, Li P-Q, Fu Q-Y, Li Q-X, Cai W-W. CYP2C19 genotype and omeprazole hydroxylation phenotype in Chinese Li population. *Clin Exp Pharmacol Physiol*; **34**: 421–424 (2007).

47 Ding Y *et al.* Genetic polymorphisms and phenotypic analysis of drug-metabolizing enzyme CYP2C19 in a Li Chinese population. *Int J Clin Exp Pathol*; **8**: 13201–8 (2015).

48 Yang ZF *et al.* Genetic polymorphisms of cytochrome P450 enzymes 2C9 and 2C19 in a healthy Mongolian population in China. *Genet Mol Res*; **9**: 1844–1851 (2010).

49 Chen L *et al.* Genetic polymorphism analysis of CYP2C19 in Chinese Han populations from different geographic areas of mainland China. *Pharmacogenomics*; **9**: 691–702 (2008).

50 Shin D-J *et al.* Association of CYP2C19*2 and *3 Genetic Variants with Essential Hypertension in Koreans. *Yonsei Med J*; **53**: 1113 (2012).

51 Kim KA, Song WK, Park JY. Association of CYP2B6, CYP3A5, and CYP2C19 Genetic Polymorphisms With Sibutramine Pharmacokinetics in Healthy Korean Subjects. *Clin Pharmacol Ther*; **86**: 511–518 (2009).

52 Ramsjö M *et al.* CYP2C19 activity comparison between Swedes and Koreans: effect of genotype, sex, oral contraceptive use, and smoking. *Eur J Clin Pharmacol*; **66**: 871–877 (2010).

53 Lee SS *et al.* Comparisons of CYP2C19 Genetic Polymorphisms Between Korean and Vietnamese Populations. *Ther Drug Monit*; **29**: 455–459 (2007).

54 Roh HK *et al.* CYP2C19 genotype and phenotype determined by omeprazole in a Korean population. *Pharmacogenetics*; **6**: 547–51 (1996).

55 Yoo HD, Park SA, Cho HY, Lee YB. Influence of CYP3A and CYP2C19 genetic polymorphisms on the pharmacokinetics of cilostazol in healthy subjects. *Clin Pharmacol Ther*; **86**: 281–284 (2009).

56 Jin T *et al.* Genotype-phenotype analysis of CYP2C19 in the Tibetan population and its potential clinical implications in drug therapy. *Mol Med Rep*; **13**: 2117–2123 (2016).

57 Jin T *et al.* Genetic polymorphisms of the drug-metabolizing enzyme CYP2C19 in the Uyghur population in northwest China. *Xenobiotica*; **46**: 634–640 (2016).

58 Mirzaev KB *et al.* Genetic Polymorphisms of Cytochrome P450 Enzymes and Transport Proteins in a Russian Population and Three Ethnic Groups of Dagestan. *Genet Test Mol Biomarkers*; **21**: 747–753 (2017).

59 Pedersen RS *et al.* Linkage disequilibrium between the CYP2C19*17 allele and wildtype CYP2C8 and CYP2C9 alleles: Identification of CYP2C haplotypes in healthy Nordic populations. *Eur J Clin Pharmacol*; **66**: 1199–1205 (2010).

60 Zhou Y, Lauschke VM. Comprehensive overview of the pharmacogenetic diversity in Ashkenazi Jews. *J Med Genet*; **55**: 617–627 (2018).

61 Tervasmäki A, Winqvist R, Jukkola-Vuorinen A, Pylkäs K. Recurrent CYP2C19 deletion allele is associated with triple-negative breast cancer. *BMC Cancer*; **14**: 1–7 (2014).

62 Ragia G, Arvanitidis KI, Tavridou A, Manolopoulos VG. Need for reassessment of reported CYP2C19 allele frequencies in various populations in view of CYP2C19*17 discovery: the case of Greece. *Pharmacogenomics*; **10**: 43–49 (2009).

63 Scott SA *et al.* Identification of CYP2C19*4B: Pharmacogenetic implications for drug metabolism including clopidogrel responsiveness. *Pharmacogenomics J*; **12**: 297–305 (2012).

64 Jakovski K, Nestorovska AK, Labacevski N, Dimovski AJ. Characterization of the most common CYP2C9 and CYP2C19 allelic variants in the population from the Republic of Macedonia. *Pharmazie*; **68**: 893–8 (2013).

65 Sychev D *et al.* Comparison of CYP2C9, CYP2C19, CYP2D6, ABCB1, and SLCO1B1 gene-polymorphism frequency in Russian and Nanai populations. *Pharmgenomics Pers Med*; **Volume10**: 93–99 (2017).

66 Dehbozorgi M *et al.* Prevalence of the CYP2C19*2 (681 G>A), *3 (636 G>A) and *17 (‑806>T) alleles among an Iranian population of different ethnicities. *Mol Med Rep*; **17**: 4195–4202 (2018).

67 Payan M, Tajik N, Rouini MR, Ghahremani MH. Genotype and allele frequency of CYP2C19*17 in a healthy Iranian population. *Med J Islam Repub Iran*; **29**: 269 (2015).

68 Hashemizadeh Z, Malek-Hosseini SA, Badiee P. Prevalence of CYP2C19 Genetic Polymorphism among Normal People and Patients with Hepatic Diseases. *Int J organ Transplant Med*; **9**: 27–33 (2018).

69 Saeed LH, Mayet AY. Genotype-Phenotype Analysis of CYP2C19 in Healthy Saudi Individuals and its Potential Clinical Implication in Drug Therapy. *Int J Med Sci*; **10**: 1497–1502 (2013).

70 Al-Jenoobi FI *et al.* CYP2C19 Genetic Polymorphism in Saudi Arabians. *Basic Clin Pharmacol Toxicol*; **112**: 50–54 (2013).

71 Arici M, Özhan G. CYP2C9, CYPC19 and CYP2D6 gene profiles and gene susceptibility to drug response and toxicity in Turkish population. *Saudi Pharm J*; **25**: 376–380 (2017).

72 Uckun Z *et al.* The impact of CYP2C19 polymorphisms on citalopram metabolism in patients with major depressive disorder. *J Clin Pharm Ther*; **40**: 672–679 (2015).
