# Supplementary figures and images for: Meta-analysis of probability estimates of worldwide variation of CYP2D6 and CYP2C19
Source: Transl Psychiatry. 2021 Feb 24;11:141. doi: 10.1038/s41398-020-01129-1 (PMC7904867; doi:10.1038/s41398-020-01129-1)

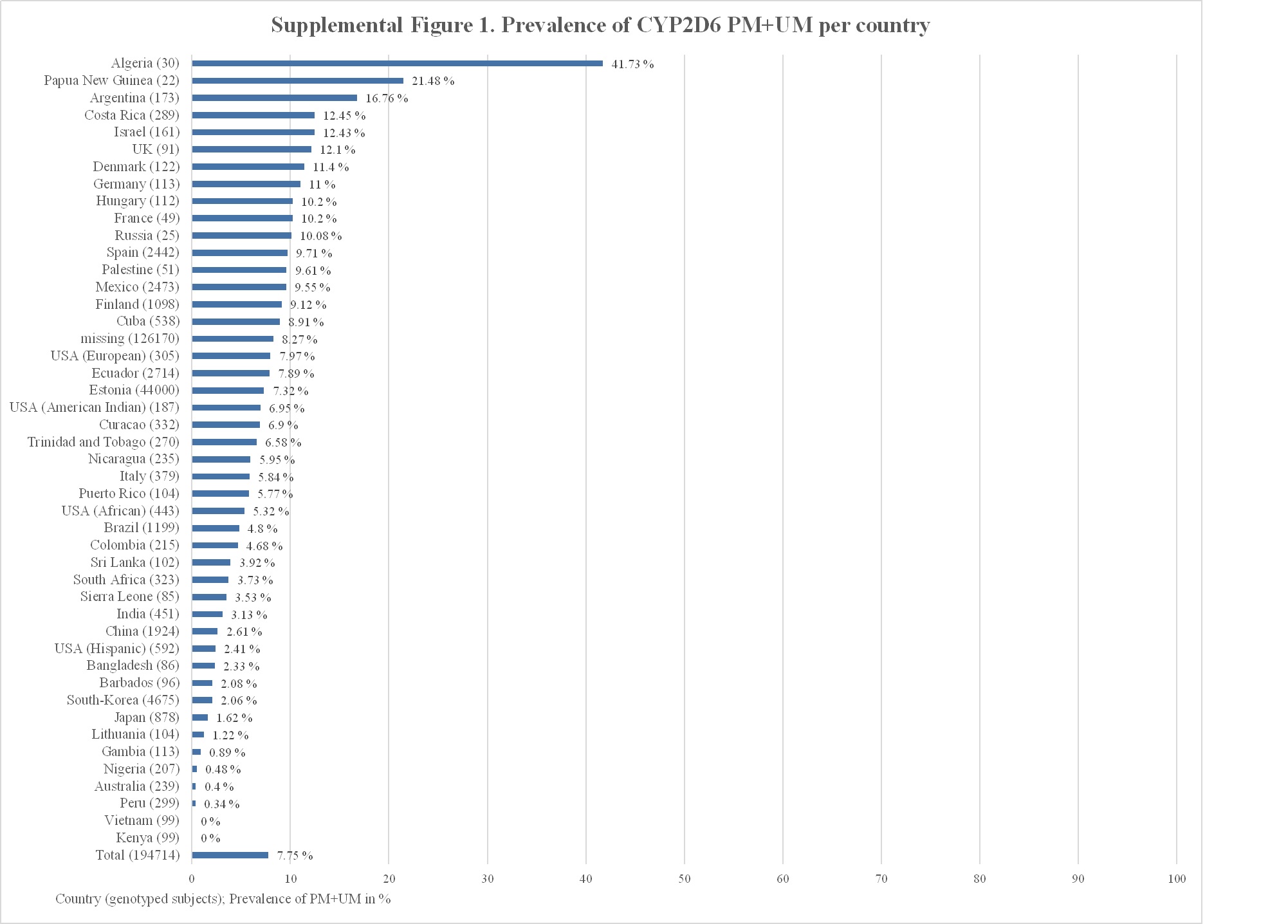

Supplement: Supplementary file 5 — Supplemental Figure 1. Prevalence of CYP2D6 PM+UM per country [file 41398_2020_1129_MOESM5_ESM.jpg]

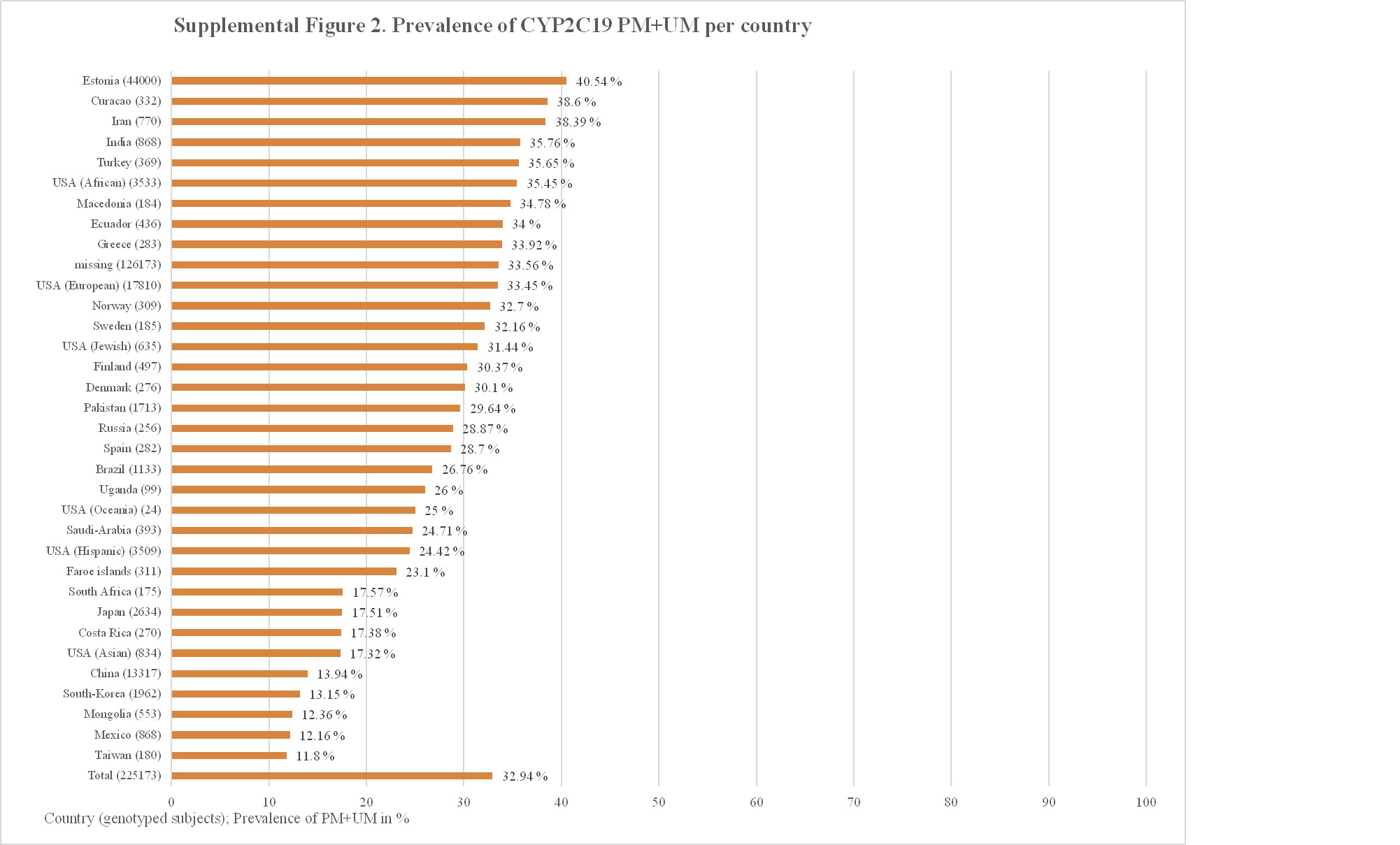

Supplement: Supplementary file 6 — Supplemental Figure 2. Prevalence of CYP2C19 PM+UM per country [file 41398_2020_1129_MOESM6_ESM.jpg]
